# Construction and validation of a model based on immunogenic cell death-associated lncRNAs to predict prognosis and direct therapy for kidney renal clear cell carcinoma

**DOI:** 10.18632/aging.204741

**Published:** 2023-06-27

**Authors:** Chenxi Cai, Kexin Shu, Wanying Chen, Jiatong Ding, Zishun Guo, Yiping Wei, Wenxiong Zhang

**Affiliations:** 1 Department of Thoracic Surgery, The Second Affiliated Hospital of Nanchang University, Nanchang 330006, China; 2 Jiangxi Medical College, Nanchang University, Nanchang 330006, China; 3 Department of Urinary Surgery, The Second Affiliated Hospital of Nanchang University, Nanchang 330006, China

**Keywords:** immunogenic cell death, long noncoding RNAs, kidney renal clear cell carcinoma, prognostic signature

## Abstract

Background: Immunogenic cell death (ICD) is an important part of the antitumor effect, yet the role played by long noncoding RNAs (lncRNAs) remains unclear. We explored the value of ICD-related lncRNAs in tumor prognosis assessment in kidney renal clear cell carcinoma (KIRC) patients to provide a basis for answering the above questions.

Methods: Data on KIRC patients were obtained from The Cancer Genome Atlas (TCGA) database, prognostic markers were identified, and their accuracy was verified. An application-validated nomogram was developed based on this information. Furthermore, we performed enrichment analysis, tumor mutational burden (TMB) analysis, tumor microenvironment (TME) analysis, and drug sensitivity prediction to explore the mechanism of action and clinical application value of the model. RT-qPCR was performed to detect the expression of lncRNAs.

Results: The risk assessment model constructed using eight ICD-related lncRNAs provided insight into patient prognoses. Kaplan-Meier (K-M) survival curves showed a more unfavorable outcome in high-risk patients (p<0.001). The model had good predictive value for different clinical subgroups, and the nomogram constructed based on this model worked well (risk score AUC=0.765). Enrichment analysis revealed that mitochondrial function-related pathways were enriched in the low-risk group. The adverse prognosis of the higher-risk cohort might correspond to a higher TMB. The TME analysis revealed a higher resistance to immunotherapy in the increased-risk subgroup. Drug sensitivity analysis can guide the selection and application of antitumor drugs in different risk groups.

Conclusions: This prognostic signature based on eight ICD-associated lncRNAs has significant implications for prognostic assessment and treatment selection in KIRC.

## INTRODUCTION

Kidney renal clear cell carcinoma (KIRC) is the most common subtype of renal cell carcinoma, constituting approximately 75%-85% of renal cell carcinoma cases [1]. Its morbidity is rising each year, and patients often present with postoperative metastases, which are associated with great patient hardship [2]. The American Joint Committee on Cancer (AJCC) tumor node metastasis (TNM) classification system is often used for staging and is used to divide patients into stages I, II, III, and IV for prognostic assessment [3]. However, the predictions of the prognosis of patients treated based on this system are not accurate [4]. Therefore, novel prognostic models are urgently needed to guide the treatment of patients with KIRC. Biomarker-based prognostic models have shown great potential in recent years for tumor patient prognostic assessment.

Immunogenic cell death (ICD), one of the important modalities of regulatory cell death, promotes antitumor effects by triggering the activation of cytotoxic T cells [5]. Simultaneously, long noncoding RNAs (lncRNAs) are indispensable for performing this function, and lncRNA-related models have been successfully constructed for numerous cancer types. lncRNA models constructed by Liu Z et al. were validated in the prognostic assessment of colorectal cancer patients [6]. lncRNA contributions in tumor therapy were reviewed by Eptaminitaki GC et al. [7]. Liang YL et al. constructed a tumor immune heterogeneity-associated lncRNA prognostic model to determine the long-term prognosis of patients with nasopharyngeal carcinoma [8]. However, a KIRC prognostic assessment model based on ICD-related lncRNAs is still not available.

Thus, we constructed an ICD-associated lncRNA-based prognostic model for assessing the prognosis of patients with KIRC and appraised its clinical application value through enrichment analysis, tumor mutational burden (TMB) analysis, tumor microenvironment (TME) differential analysis, and drug sensitivity prediction.

## MATERIALS AND METHODS

### Data sources and access

The University of California, Santa Cruz (UCSC) Xena database (https://xena.ucsc.edu/, until November 1st, 2022) was the primary source of data for this study [9]. The Cancer Genome Atlas (TCGA) database in Xena provided patient information, including transcriptomic data for 607 KIRC cases and clinicopathological information for 979 KIRC clinical patients. Tumor somatic cell mutation data were obtained from the TCGA database (https://portal.gdc.cancer.gov/repository, until November 1st, 2022). Transcriptomic data were obtained in two data formats, HTSeq-Counts and HTSeq-FPKM, which are used for different types of data analysis. We selected patients with complete transcriptional and clinical information and ensured that they were identified in both datasets. Ultimately, a total of 597 samples were analyzed in this study.

### Select ICD-associated genes and ICD-related lncRNAs

ICD-associated genes were identified from the GeneCards website (https://www.genecards.org/, until November 1st, 2022). We conducted a search using immunogenic cell death as a keyword, and target genes were selected for subsequent analysis [10]. In addition, we conducted Pearson correlation analysis to investigate the associations among ICD-associated genes and all lncRNAs and acquired ICD-related lncRNAs (filter conditions indicated as |correlation coefficient| > 0.3 and p < 0.05). We ran variance analysis on the results obtained from Pearson correlation analysis using the “DESeq2” package [11], with the retention of lncRNAs with a p value < 0.05 and |log2-fold change > 3|.

All KIRC patients were randomly assigned 1:1 to the training cohort, which was used for model construction, or the test cohort, which was used to verify the model. Then, ICD-associated lncRNAs relevant to patient survival were short-listed in the training cohort using univariate Cox regression analysis. We used least absolute shrinkage and selection operator (LASSO) regression analysis to avoid overfitting. Finally, ICD-related lncRNAs were identified through multivariate Cox regression analysis.

### KIRC ICD-associated lncRNA prognostic model

We used the results from predictive model building. The format of the risk score was as follows: risk score = coefficient (lncRNA_1_) × expression (lncRNA_1_) + coefficient (lncRNA_2_) × expression (lncRNA_2_) + coefficient (lncRNA_3_) × expression (lncRNA_3_) +......+ coefficient (lncRNA_n_) × expression (lncRNA_n_). Using the midpoint of the risk score as its threshold, the training group, the test group and all patients were sorted into high- and low-risk cohorts for survival analysis. Kaplan-Meier (K-M) survival analysis utilizing “survival” and “survminer” was conducted to examine the variation in overall survival (OS) between risk categories in the training cohort, test cohort, and total cohort. Receiver operating characteristic (ROC) values at one, three, and five years were extrapolated to evaluate the predictive performance of the signature.

Principal component analysis (PCA) using the expression profiles of all genes, all lncRNAs, lncRNAs associated with ICD, and lncRNAs in the selected prognostic models was conducted to validate the subgrouping effect. In addition, univariate and multifactor Cox independent prognostic analyses were conducted to assess risk scores and clinical data to validate the predictive value of the risk model. After that, K-M survival analysis was conducted to probe the model feasibility under dissimilar clinical characteristics.

### Creation of a nomogram for predicting patient survival

We used the R programming language “RMS” to create a prognostic nomogram using age, risk group, and tumor stage as factors to predict patient outcomes at 1, 3, and 5 years [12]. We also determined the value of this nomogram in prognosis prediction.

### Enrichment analysis

KEGG enrichment analysis was applied to optimize the functions that might be interrelated with the genetic set. We divided the cases into high- and low-risk groups under the simulations constructed from 8 ICD-related lncRNAs, and enrichment analysis was used to filter the pathways with a p value<0.05 and false discovery rate (FDR)<0.25 using GSEA_4.3.2 software (downloaded at https://www.gsea-msigdb.org) [13].

### Tumor mutational burden contributes to the prognostic evaluation of tumors

We obtained tumor somatic mutation data from the TCGA database. We manipulated the data using the “TCGAbiolinks” package, sketched waterfall plots using the R programming language “maftools”, and calculated TMB [14, 15].

### Evaluation of TME and immuno-infiltration

The R software package “ESTIMATE” was used as an analysis vehicle to calculate any difference in stromal score, ESTIMATE score, immune score, and tumor purity between the high- and low-risk subgroups. We obtained further information through the Tumor Immune Estimation Resource (TIMER) 2.0 website (http://timer.cistrome.org/, until November 1st, 2022) using “TIMER”, “CIBERSORT”, “CIBERSORT-ABS”, “QUANTISEQ”, “XCELL”, and “EPIC”, together with “MCPCOUNTER”, which are six methods used to optimize the correlation between individual immune cell types and risk scores [16]. To detail the constitutive shifts in immune cells, we undertook an immune infiltration analysis of 22 immune cell lineages.

### ICD-related signature in immunotherapy versus chemotherapy

To fully address the distinctions between KIRC patients in different risk groups presenting with tumor immune dysfunction and rejection, the Tumor Immune Dysfunction and Exclusion (TIDE) website (http://tide.dfci.harvard.edu/, until November 1st, 2022) was used to retrieve the TIDE scores and related data of KIRC patients [17]. Therefore, we conducted single-sample gene set enrichment analysis (ssGSEA) using the “GSVA” R package. Half-maximal inhibitory concentrations (IC50) of typical antineoplastic drugs were determined using the R software “oncoPredict” package [18]. The IC50 values were compared between different classes utilizing a Wilcoxon signed-rank test.

### Validation by RT-qPCR

Twenty-one KIRC samples were obtained from the Second Affiliated Hospital of Nanchang University, and patients were divided into high- and low-risk groups. The study was approved by the Ethics Committee of the Second Affiliated Hospital of Nanchang University, and all participants gave their informed consent. We extracted RNA from each KIRC tissue sample using TRIzol reagent (Life Technologies CA, USA) and randomly selected samples for RT-qPCR analysis. The experiments were performed using BlazeTaq SYBR Green qPCR master mix (GeneCopoeia, Guangzhou, China) and the Applied Biosystems 7500 Fast Real-Time PCR System (Applied Biosystems). All RNAs of every sample were analyzed in three independent experiments. The primers for ICD-associated lncRNAs are shown in Supplementary Table 1. The relative expression of lncRNAs was calculated using the 2^(-ΔΔCt) method.

We used the Human Protein Atlas (https://www.proteinatlas.org/) database to compare the protein expression levels of selected ICD-related genes in KIRC tissues with those in normal tissues.

### Availability of data and material

The data sets used and/or analyzed during the current study are available from the corresponding author upon reasonable request.

## RESULTS

### Screening for ICD-associated lncRNAs in KIRC patients

**Figure 1:**
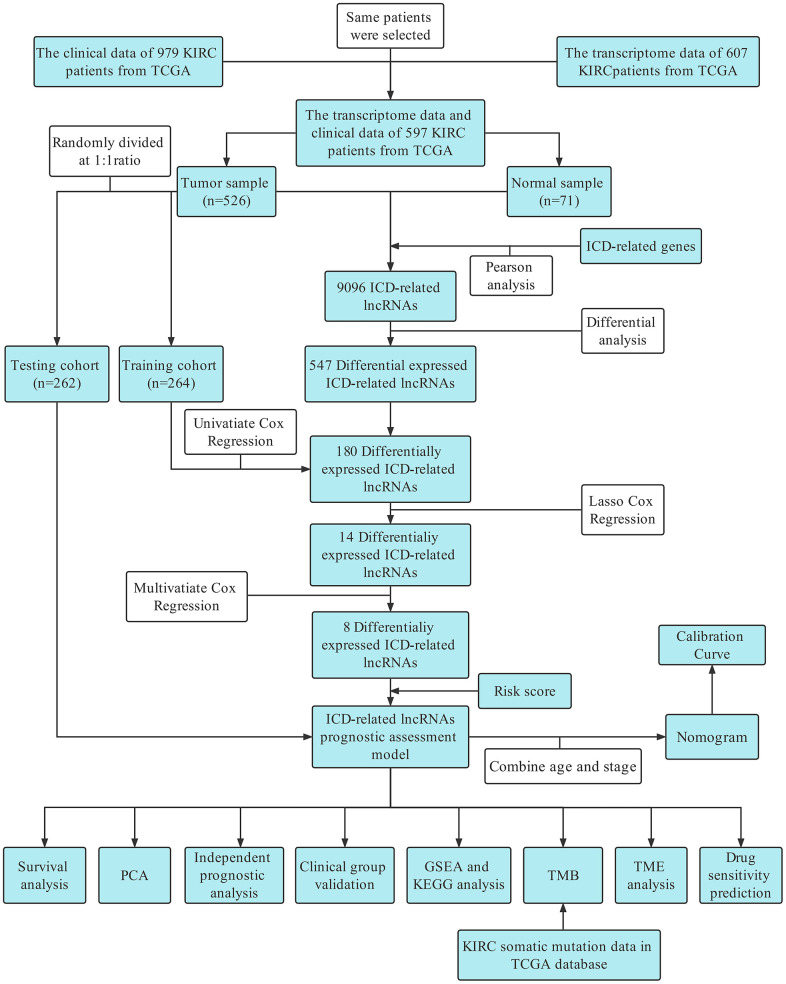
Flow chart.

**Figure 2:**
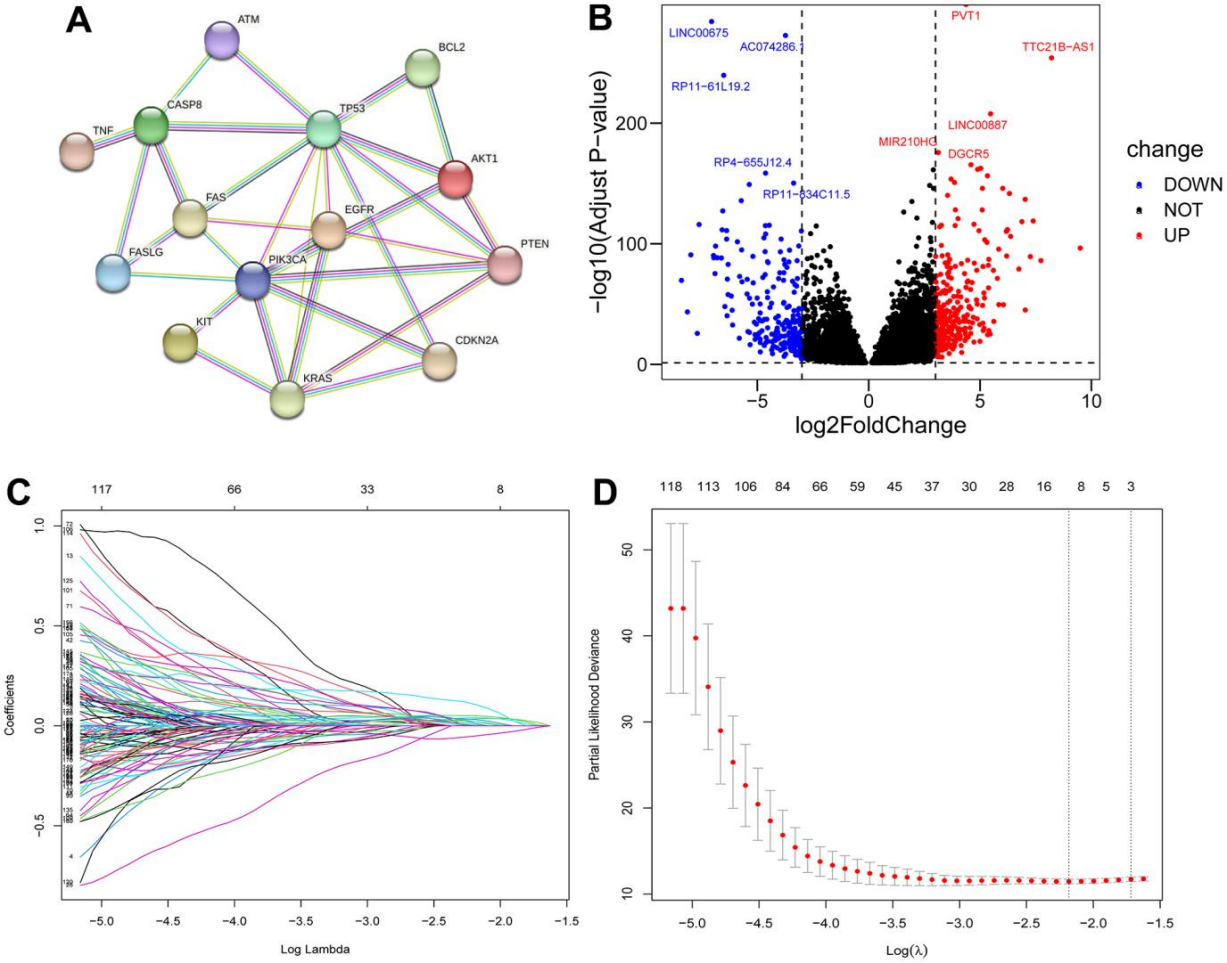
**Filter for ICD-related lncRNAs.** PPI network among 16 ICD-related genes (**A**); Volcano plot of differentially expressed ICD-associated lncRNAs (**B**); LASSO regression analysis (**C**, **D**).

The overall study design of this paper is displayed in a flow chart (Figure 1). First, we identified ICD-related genes and constructed a protein–protein interaction (PPI) network associated with 16 of these genes by using the STRING website (Figure 2A). Further Pearson correlation analysis was executed, from which we derived 9096 ICD-associated lncRNAs. Next, we narrowed the pool down to 547 lncRNAs that were shown by differential analysis to be differentially expressed in tumor tissues (Figure 2B).

Based on difference analysis, we rerandomized 526 patients into training and test groups (Table 1). In the training group, a single-factor Cox model was used to screen 180 lncRNAs associated with differential prognosis using p<0.05 as the cutoff value (Supplementary Table 2). Follow-up LASSO analysis revealed 14 differentially expressed ICD-related lncRNAs (Figure 2C, 2D) (Supplementary Table 3), and multifactorial Cox regression analysis was used to further screen the 8 most significant lncRNAs (Supplementary Figure 1A). We subsequently explored the correlations among these 8 lncRNAs (Supplementary Figure 1B) and their correlation with ICD-related genes (Supplementary Figure 1C) that were found to exhibit a marked difference in up- and downregulation in tumor tissues (Supplementary Figure 1D).

**Table 1:** Clinical information of the patients in the test and training groups.

**Characteristics**	**Train cohort (n=264)**			**Test cohort (n=262)**			**Entire cohort (n=526)**	
	**n**	**%**		**n**	**%**		**n**	**%**
**Age**								
<65	184	69.70		163	62.21		347	65.97
>65	80	30.30		99	37.79		179	34.03
**Status**								
Alive	176	66.67		179	68.32		355	67.49
Dead	88	33.33		83	31.68		171	32.51
**Gender**								
Female	90	34.10		93	35.50		183	34.79
Male	174	69.90		169	64.50		343	65.21
**Stage**								
Stage I	128	48.48		133	50.76		261	49.62
Stage II	30	11.36		27	10.31		57	10.84
Stage III	62	23.48		61	23.28		123	23.38
Stage IV	42	15.91		40	15.27		82	15.59
Unknow	2	0.76		1	0.38		3	0.57
**T stage**								
T1	132	50.00		135	51.53		267	50.76
T2	36	13.64		33	12.60		69	13.12
T3	91	34.47		88	33.59		179	34.03
T4	5	1.89		6	2.29		11	2.09
**M stage**								
M0	209	79.17		209	79.77		418	79.47
M1	42	15.91		36	13.74		78	14.83
Unknow	13	4.92		17	6.49		30	5.70
**N stage**								
N0	119	45.08		119	45.42		238	45.25
N1	8	0.03		8	3.05		16	3.04
Unknow	137	51.89		135	51.53		272	51.71
**Race**								
White	232	87.88		225	85.88		457	86.88
Black or African American	25	9.47		29	11.07		54	10.27
Asian	3	1.14		5	1.91		8	1.52
Unknow	4	1.52		3	1.15		7	1.33

Abbreviation: T stage, Tumor stage; N stage, Node stage; M stage, metastasis stage.

### Building and validating the prognostic model

**Figure 3:**
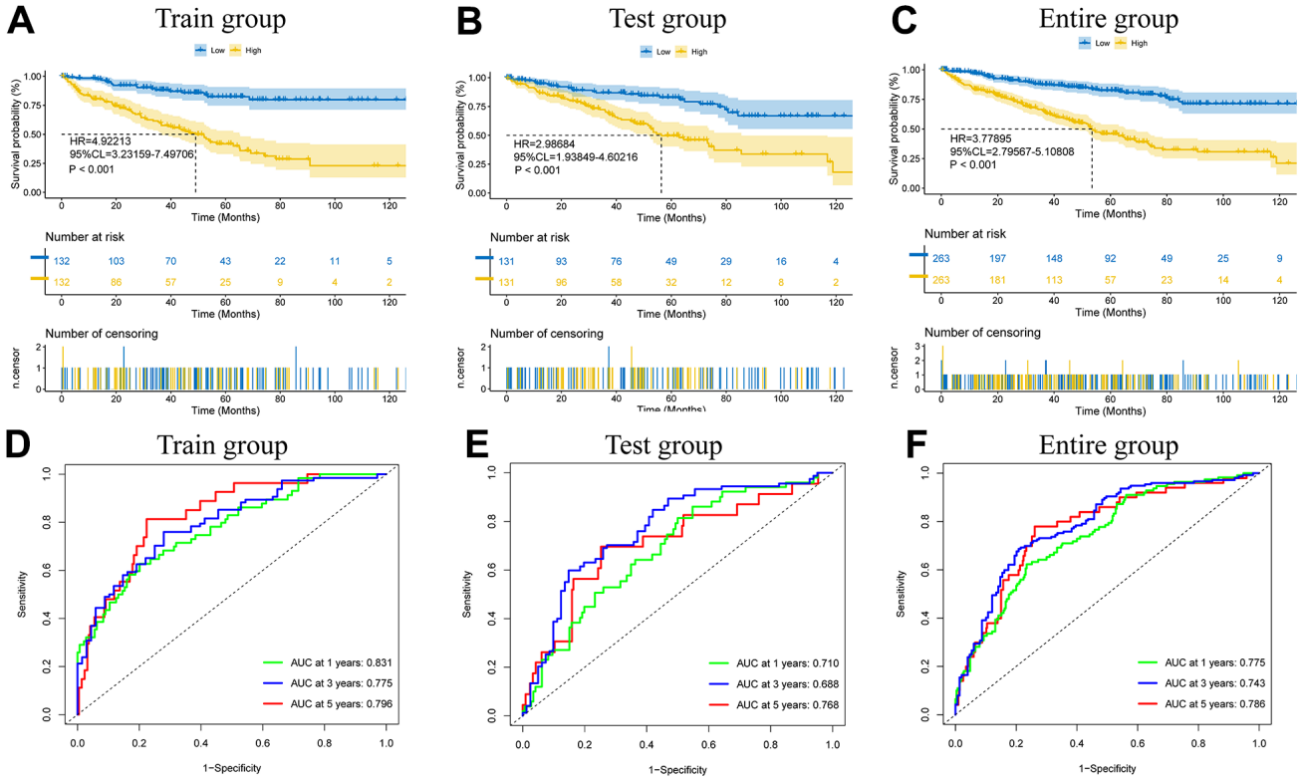
**The model prediction effect is validated by the training group, test group, and entire group.** K-M analysis (**A**–**C**) and Time-dependent ROC curves (**D**–**F**) to compare the survival of the high-risk group and low-risk group.

Based on the 8 ICD-related lncRNAs, we calculated risk scores for different patients: risk score= coefficient(*AP000439.3*)×expression (*AP000439.3*)+ coefficient (*RP11.1151B14.5*)×expression(*RP11.1151B14.5*)+coefficient(*RP11.479J7.2*)×expression(*RP11.479J7.2*)+coefficient(*AC099552.4*)×expression(*AC099552.4*)+coefficient(*RP11.19E11.1*)×expression(*RP11.19E11.1*)+coefficient(*CTB.33O18.1*)×expression(*CTB.33O18.1*)+coefficient(*RP11.339D23.1*)×expression(*RP11.339D23.1*)+coefficient(*LINC01192*)×expression(*LINC01192*). Clinical information for all patients in the high- and low-risk clusters is presented in Table 2. In the training cohort, the test cohort, and the total cohort, survival was much worse in the high-risk group than in the low-risk group (all p values<0.001) (Figure 3A–3C), and the time-dependent ROC curve demonstrated that the model’s predictive value was excellent (AUCs were 0.831, 0.775, and 0.796 for the training cohort at one, three, and five years, respectively; 0.710, 0.688, and 0.768 for the test cohort at one, three, and five years, respectively; and 0.775, 0.743, and 0.786 for the total cohort at one, three, and five years, respectively) (Figure 3D–3F). In different cohorts, the manifestations of 8 lncRNAs in patients with different risks (Supplementary Figure 2A–2C), the risk curves (Supplementary Figure 2D–2F), the risk distribution plot (Supplementary Figure 2G–2I), and the scatter plot (Supplementary Figure 2J–2L) showed the forecasting value of the model.

**Table 2:** Clinical information for 526 patients in different risk categories.

**Characteristics**	**High-risk group (n=263)**			**Low-risk group (n=263)**	
	**n**	**%**		**n**	**%**
**Age**					
<65	163	61.98		184	69.96
>65	100	38.02		79	30.04
**Status**					
Alive	134	50.95		221	84.03
Dead	129	49.05		42	15.97
**Gender**					
Female	90	34.22		93	35.36
Male	173	65.78		170	64.64
**Stage**					
Stage I	100	38.02		161	61.22
Stage II	27	10.27		30	11.41
Stage III	74	28.14		49	18.62
Stage IV	60	22.81		22	8.37
Unknow	2	0.76		1	0.38
**T stage**					
T1	104	39.54		163	61.98
T2	36	13.69		33	12.55
T3	113	42.97		66	25.10
T4	10	3.80		1	0.38
**M stage**					
M0	192	73.10		226	85.93
M1	57	21.67		21	7.98
Unknow	14	5.32		16	6.08
**N stage**					
N0	132	50.19		106	40.30
N1	13	4.94		3	1.14
Unknow	118	44.87		154	58.56
**Race**					
White	226	85.93		231	87.83
Black or African American	29	11.03		25	9.51
Asian	4	1.52		4	1.52
Unknow	4	1.52		3	1.14

Abbreviation: T stage, Tumor stage; N stage, Node stage; M stage, metastasis stage.

**Figure 4:**
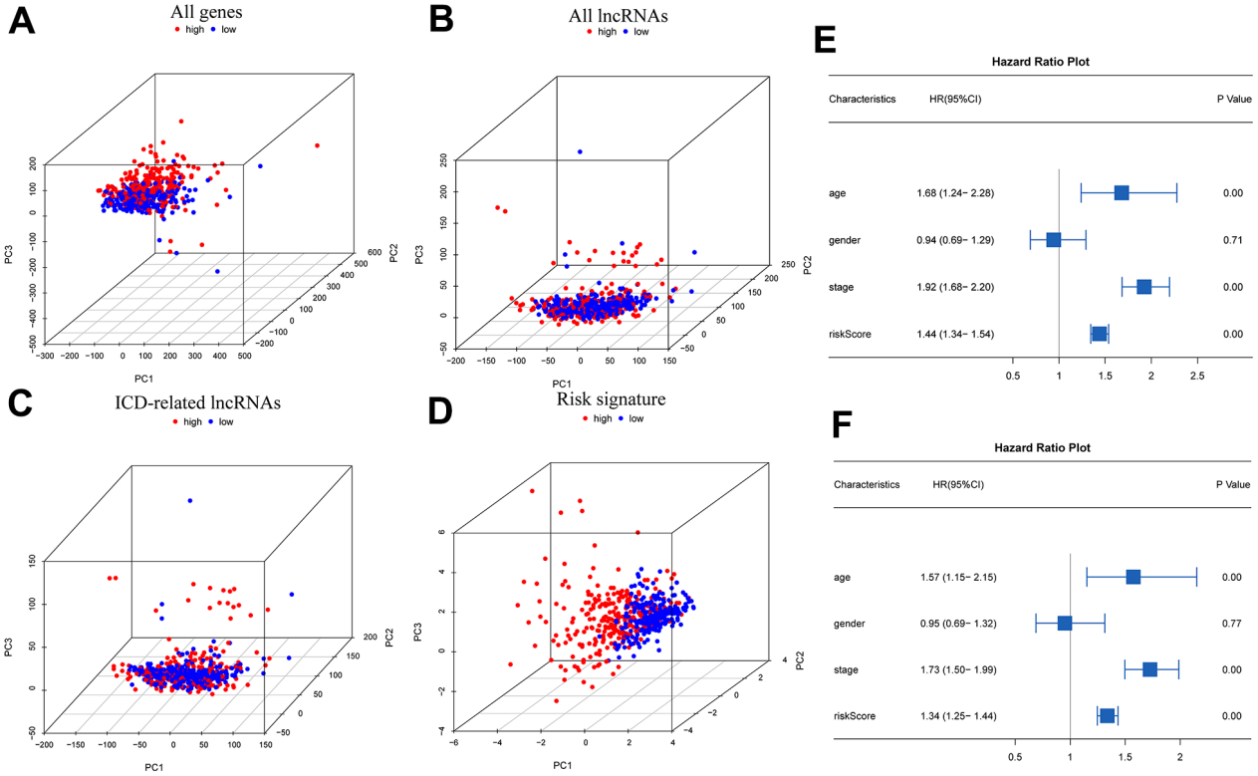
**PCA and independent prognostic analysis of the signature.** PCA based on all genes (**A**), all lncRNAs (**B**), ICD-related lncRNAs (**C**), and risk signature (**D**); Univariate (**E**) and multivariate (**F**) independent prognostic analysis.

The outcomes of principal component analysis (PCA) (Figure 4A–4D) indicated that the high- and low-risk subgroups showed obvious categorical clustering. Univariate Cox independent prognostic analysis indicated that age (HR=1.68, 95% CL=1.24-2.28, p<0.001), tumor stage (HR=1.92, 95% CL=1.68-2.20, p<0.001), and risk score (HR=1.44, 95% CL=1.34-1.54, p<0.001) were standalone risk factors (Figure 4E). Likewise, multifactorial independent prognostic analysis showed that age (HR=1.57, 95% CL=1.15-2.15, p=0.004), tumor stage (HR=1.73, 95% CL=1.50-1.99, p<0.001), and risk score (HR=1.34, 95% CL=1.25-1.44, p<0.001) were standalone risk factors (Figure 4F).

**Figure 5:**
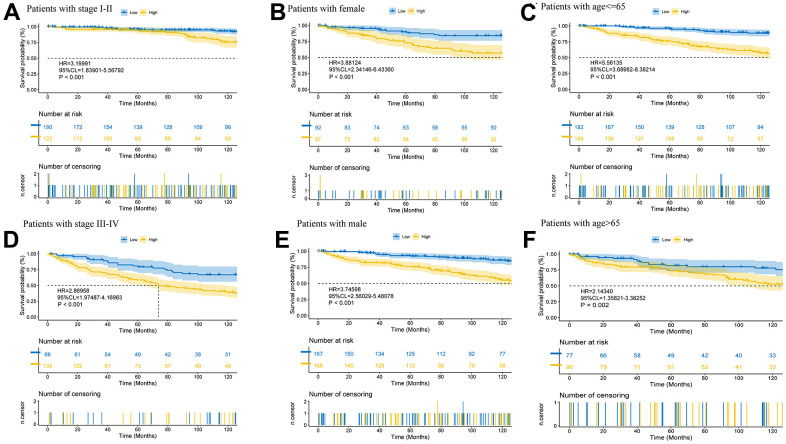
**Further validation of model effects.** Survival curves of patients in different clinical states (**A**–**F**).

The expression heatmap containing different clinical data, risk groupings, and prognostic model-related lncRNAs (Supplementary Figure 3A) and the survival curves of patients in a variety of clinical states (Figure 5) illustrate the significance of the model predictions (stage I-II: p<0.001, stage III-IV: p<0.001; female: p<0.001, male: p<0.001; age<=65: p<0.001, age>65: p=0.002).

### Clinical OS prediction nomogram

**Figure 6:**
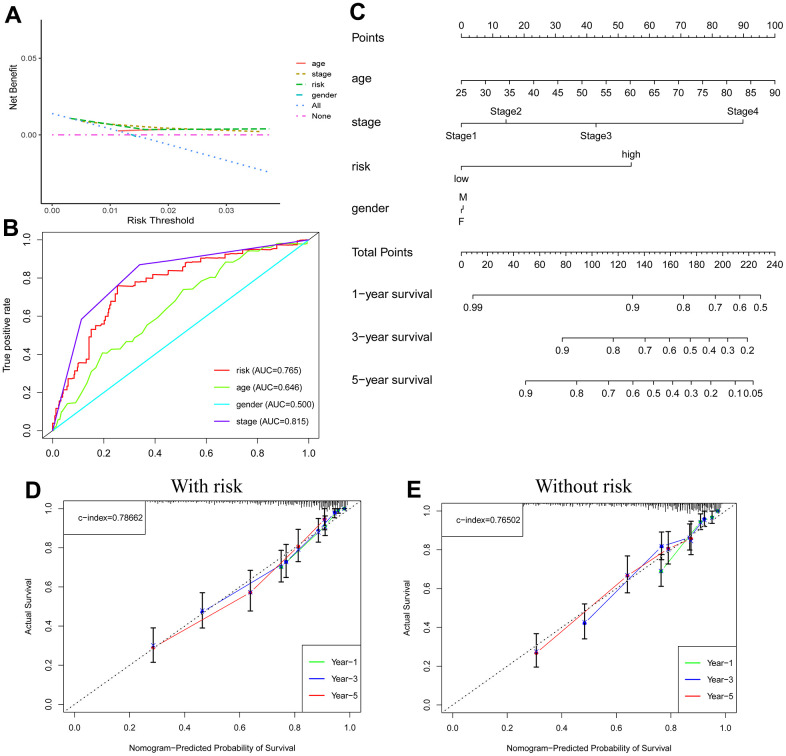
**Nomogram predicts patient prognosis.** Decision curve to test for forecast value (**A**); ROC curves containing different clinical information (**B**); A clinical prognosis nomogram is constructed by age, gender, risk, and stage together (**C**). Nomogram with (**D**) and without (**E**) risk model.

The scatter plot showed a positive correlation with respect to tumor stage and risk scores (p<0.001) (Supplementary Figure 3C). Age >65 years also tended to be a factor for an increased risk score (p=0.096) (Supplementary Figure 3B). Decision curve analysis (DCA) (Figure 6A) and ROC curves (Figure 6B) (risk AUC=0.765, age AUC=0.646, sex AUC=0.500, and stage AUC=0.815) showed that risk had a superior prediction value compared to most clinical information. A prospective estimator of KIRC patients based on age, risk, sex, and disease staging was conceived (Figure 6C), patients were used to verify its effectiveness, and the results indicated good performance (Supplementary Figure 4). The forecasting value of the nomogram combined with the risk model (Supplementary Figure 4D) was higher than that of the nomogram without the risk model (Supplementary Figure 4E).

### Enrichment analysis

KEGG enrichment analysis revealed the related functions of variably expressed genes, including ubiquitin-mediated proteolysis, glycolipid biosynthesis, galactose metabolism, ethoxylate, and dicarboxylate metabolism (Supplementary Figure 5). GSEA demonstrated that different pathways were enriched in different gene sets. Pathway functions enriched in the low-risk category in the GOBP library included mitochondrial gene expression, assembly of the mitochondrial respiratory chain complex, mitochondrial translation, neurotransmitter reuptake, and mitochondrial electron transport from NADH to ubiquinone (Supplementary Figure 6). All the pathway information is shown in Supplementary Table 4.

### Tumor mutational burden

**Figure 7:**
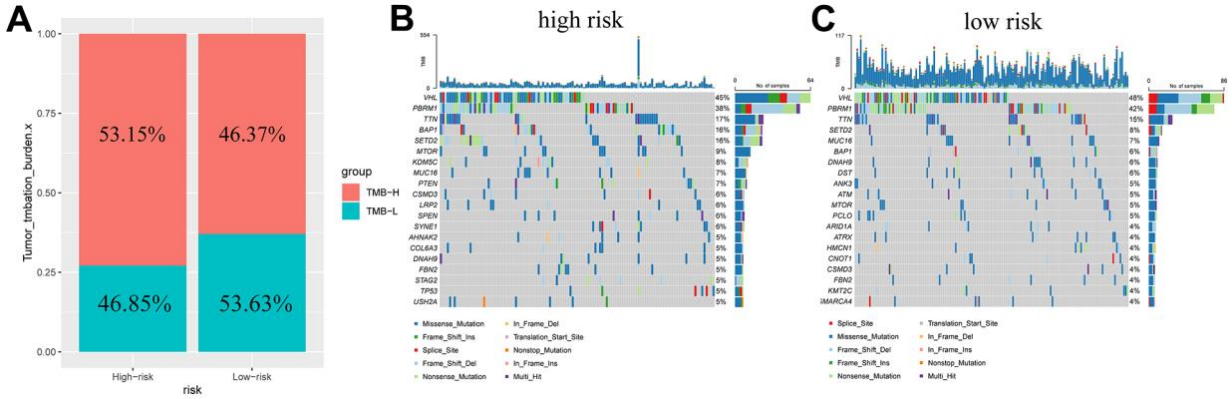
**Tumor mutation burden in different risk groups.** Percentage bar graph showing TMB for different risk subgroups (**A**); High-risk group waterfall chart (**B**); Low-risk group waterfall chart (**C**).

The TMB in patients was determined and found to be higher in the high-risk subgroup (Figure 7A). Based on this, we generated a waterfall plot of the top 20 mutant genes according to different subclusters (Figure 7B, 7C). The five most commonly mutated genes in the high-risk patients were *VHL* (45%), *PBRM1* (38%), *TTN* (17%), *BAP1* (16%), and *SETD2* (16%). *VHL* (48%), *PBRM1* (42%), *TTN* (15%), *SETD2* (8%) and *MUC16* (7%) were prone to mutation in low-risk patients.

### Tumor immune infiltration status analysis

**Figure 8:**
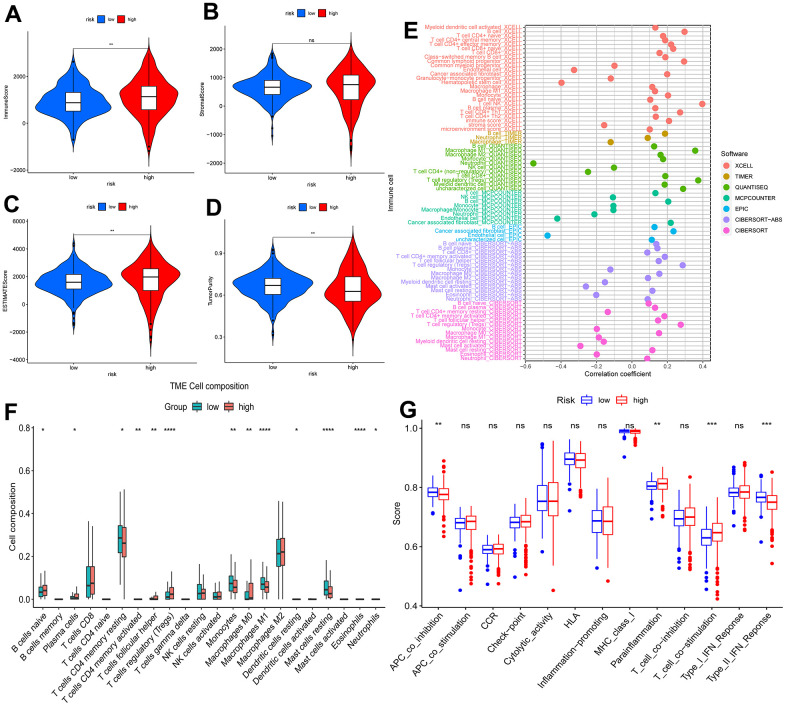
**Analysis of tumor immune microenvironment.** Violin plots of differences in immune scores (**A**), stromal scores (**B**), ESTIMATE scores (**C**), and tumor purity (**D**) for different risk subgroups; Bubble plots of correlations between immune cells and risk scores under six algorithms (**E**); Proportions of 22 immune cells in two subgroups under the CIBERSORT algorithm (**F**); single sample gene set enrichment analysis (**G**). *p < 0.05, **p < 0.01, ***p < 0.001.

The TME analysis suggested that the immune score (Figure 8A) and the ESTIMATE score (Figure 8C) were higher in the high-risk segment (p<0.01), but the stromal score (Figure 8B) did not show dramatic differences. Patients at high risk had significantly lower tumor purity (p<0.01) (Figure 8D). We hypothesized that an immunosuppressive microenvironment was present in the high-risk subgroup that weakened antitumor immunity. Additionally, we showed an association between immune cells and the risk score under different algorithms (Figure 8E). Naive B cells (p<0.05), plasma cells (p<0.05), follicular helper T cells (p<0.01), regulatory T cells (Tregs) (p<0.0001), and M0 macrophages (p<0.01) constituted a greater proportion within the high-risk group, and resting memory CD4 T cells (p<0.05), monocytes (p<0.01), M1 macrophages (p<0.0001), and resting mast cells (p< 0.0001) showed comparatively higher expression in the low-risk group (Figure 8F). The relationships among immune cells and risk scores are shown in Supplementary Figure 7. Accordingly, APC coinhibition (p<0.01) and type II IFN response (p<0.001) functions were inhibited in high-risk KIRC patients, while parainflammation (p<0.01) and T-cell costimulation functions were improved (Figure 8G). It can be speculated that tumor development in high-risk KIRC patients is facilitated by both T-cell parainflammation and costimulation.

### Benefits of promotional models in the treatment of KIRC

T-cell exclusion and T-cell dysfunction, together with TIDE scores, were consistently higher in the high-risk patients with ICD-related signatures (Supplementary Figure 8). This may indicate that there is more potential for tumors in the high-risk subgroup to exhibit immune escape, complicating treatment. The antitumor drugs with different mechanisms of action to which tumors in the low-risk subgroup are sensitive are shown in Supplementary Table 5, those to which tumors in the higher-risk subgroup are sensitive are shown in Supplementary Table 6, and those without significant intergroup susceptibility differences are shown in Supplementary Table 7. The high-risk group appears to be more sensitive to drugs that act on the PI3K/mTOR signaling pathway and metabolism pathway, which can guide drug selection.

### *In vitro* experimental validation of risk models

The protein expression levels of selected ICD-related genes in KIRC tissues and normal tissues were visualized with immunohistochemical staining images in the HPA database (Supplementary Figure 9A).

RT-qPCR results showed that among the eight ICD-related lncRNAs, *AP000439.3* was highly expressed in the low-risk group, and the remaining seven lncRNAs were highly expressed in the high-risk group (Supplementary Figure 9B).

## DISCUSSION

KIRC is one of the major pathological types of renal cell carcinoma, which often spreads and metastasizes [1]. Nevertheless, the etiology and pathogenesis of this tumor remain to be further explored [19]. Hence, it is particularly important to accurately assess a patient’s prognosis and provide appropriate treatment. However, clinical TNM staging is not able to accurately assess the prognosis of patients with KIRC [4]. Accordingly, there is a great need to construct a new prognostic assessment model. ICD is an important component of regulated cell death and is involved in the antitumor process [5]. Thus, we screened ICD-related lncRNAs for the construction of a KIRC prognostic assessment model and explored their possible molecular mechanisms and clinical applications. The outcome indicated that the risk score derived from 8 ICD-associated lncRNAs could be used as a standalone predictive factor and that patients in the high-risk subgroup had a worse prognosis. The nomogram constructed in accordance with this had good predictive value. Enrichment analysis showed that mitochondria-associated pathways might be relevant in the low-risk subgroup. The model provides a reference for antitumor drug selection for KIRC patients.

ICD involves the release of danger-associated molecular patterns (DAMPs) from apoptotic tumor cells, which activate immune cells, thus promoting the antitumor effect of immune cells [5]. ICD-based prognostic models have good predictive value in other cancer types. Cai J used an ICD-based evaluation model to verify the prognosis of low-grade glioma patients with good results [20], and an ICD-related prognostic model constructed by Ma J. and team to forecast the prognosis of patients suffering from hepatocellular carcinoma also achieved satisfactory results [21]. We downloaded sample data from TCGA-KIRC patients, performed Pearson correlation analysis to identify ICD-related genes, and then carried out difference analysis to select test groups from the obtained tumor samples to conduct subsequent one-way Cox regression analysis, LASSO regression analysis, and multifactor regression analysis to obtain ICD-related lncRNAs to construct prognostic models. A nomogram was constructed to forecast the 1-, 3-, and 5-year survival rates of patients. The survival rates of patients in the ICD-related high-risk subgroup according to the prognostic model were all worse than those of patients in the ICD-related low-risk subgroup, while the risk scores had a better predictive effect than traditional tumor staging, which we speculated was due to the worse ICD effect in the upper-risk subgroup, leading to a worse prognosis. When the risk scores were applied, the nomogram predictions were better. Among ICD-related lncRNAs, *AP000439.3* is regulated by estrogen receptor (ER) and can regulate *CCND1* expression through enhancement of estrogen receptors, thereby inhibiting cell cycle progression and cell proliferation [22]. In contrast, *LINC01192* expression is upregulated in triple-negative breast cancer and is associated with the low survival likelihood of patients with triple-negative breast cancer [23]. This may give better predictive value to the risk score. Overall, our ICD-associated lncRNA prognostic model showed good predictive properties in KIRC patients and had more potential than conventional assessment methods.

We carried out GSEA and TMB analysis to detect the possible mechanisms involved. GSEA revealed numerous pathways related to mitochondrial function enriched in the low-risk subgroup. It has been shown that the enzyme RIPK3 can mediate signaling between mitochondria and the immune system to initiate antitumor immunity [24]. Impaired oxidative phosphorylation due to mitochondrial defects leads to cellular carcinogenesis [25]. More vigorous aerobic glycolysis in cancerous tissues provides energy to the tissue [26]. Additionally, a higher TMB usually results in a worse prognosis for patients with KIRC [27].

We further explored the contribution of this prognostic model to clinical drug selection. It has been shown that plasma cells can produce immunoglobulins and inhibit cell growth in the early stages of disease. At an early stage, pathological IgG can enter tumor cells through the AP2 complex and degrade overexpressed proteins through the TRIM21-mediated ubiquitin pathway, thus achieving antitumor effects [28]. M1 isoforms of tumor-associated macrophages (TAMs) can enhance antitumor immunity, and mast cell resting tends to lead to a better prognosis in KIRC patients [29, 30]. The higher content of the above cells in the low-risk subgroup may prolong patient survival. Tregs can use CTLA4 to inhibit the costimulatory signaling molecules CD80 and CD86, secrete suppressive cytokines, and directly kill effector T cells, creating an immunosuppressive microenvironment [31]. High Treg expression in high-risk groups may cause worse patient prognosis. All these immune cells point the way to immunotherapy. Additionally, the high-risk subgroup had higher sensitivity to drugs targeting the PI3K/mTOR pathway. Cellular responses dominated by the PI3K/AKT/mTOR pathway are frequently seen in KIRC and are associated with tumor progression [32]. This might be connected to the higher sensitivity of the high-risk group. Therefore, this model is expected to be useful in the selection of antineoplastic drugs for KIRC patients.

We consider the following advantages of our study. We did not find other studies reporting an ICD-associated lncRNA model that had been successfully developed to predict the outcome of KIRC patients. Nevertheless, to verify the validity of our model, we compared it with other similar studies. First, the autophagy-related lncRNA model constructed by Xuan Y et al. showed predictive value in KIRC patients, but we further calculated the tumor mutational load for our constructed model and explored its use in applications such as antitumor drug selection [33]. Second, compared with the focal death-related lncRNA model constructed by Zhou X et al., we validated our model more comprehensively using all patients, and the results were more convincing [34]. Third, the prognostic model we constructed (AUC=0.765) was more meaningful than the metastasis-related lncRNA prognostic model constructed by Dou Q et al. (AUC=0.755) for the prognostic assessment of KIRC [35]. We must acknowledge that our study still has shortcomings. The information in this study was obtained from one of many databases and was not validated with external data. Thus, further clinical experiments are needed to validate this study in the future.

## CONCLUSIONS

Overall, we developed a prognostic model with eight ICD-associated lncRNAs and constructed a nomogram, which was shown to be a valuable guide in prognostic assessment of and treatment selection for KIRC. The inferior prognosis in the high-risk cohort may be correlated with mitochondria-associated pathways and higher TMB. Due to various shortcomings, this study awaits further basic research to explore the relevant mechanisms and clinical control studies to clarify the value of the model in drug selection. Furthermore, the results of the prognostic model based on clinical samples need to be validated in further trials.

## Supplementary Material

Supplementary Figures

Supplementary Tables
