# Supplementary material for: Construction and validation of a model based on immunogenic cell death-associated lncRNAs to predict prognosis and direct therapy for kidney renal clear cell carcinoma
Source: Aging (Albany NY). 2023 Jun 27;15(12):5304–38. doi: 10.18632/aging.204741 (PMC10333057; doi:10.18632/aging.204741)
Supplement: Supplementary Figures [file aging-15-204741-s001.pdf]

SUPPLEMENTARY FIGURES

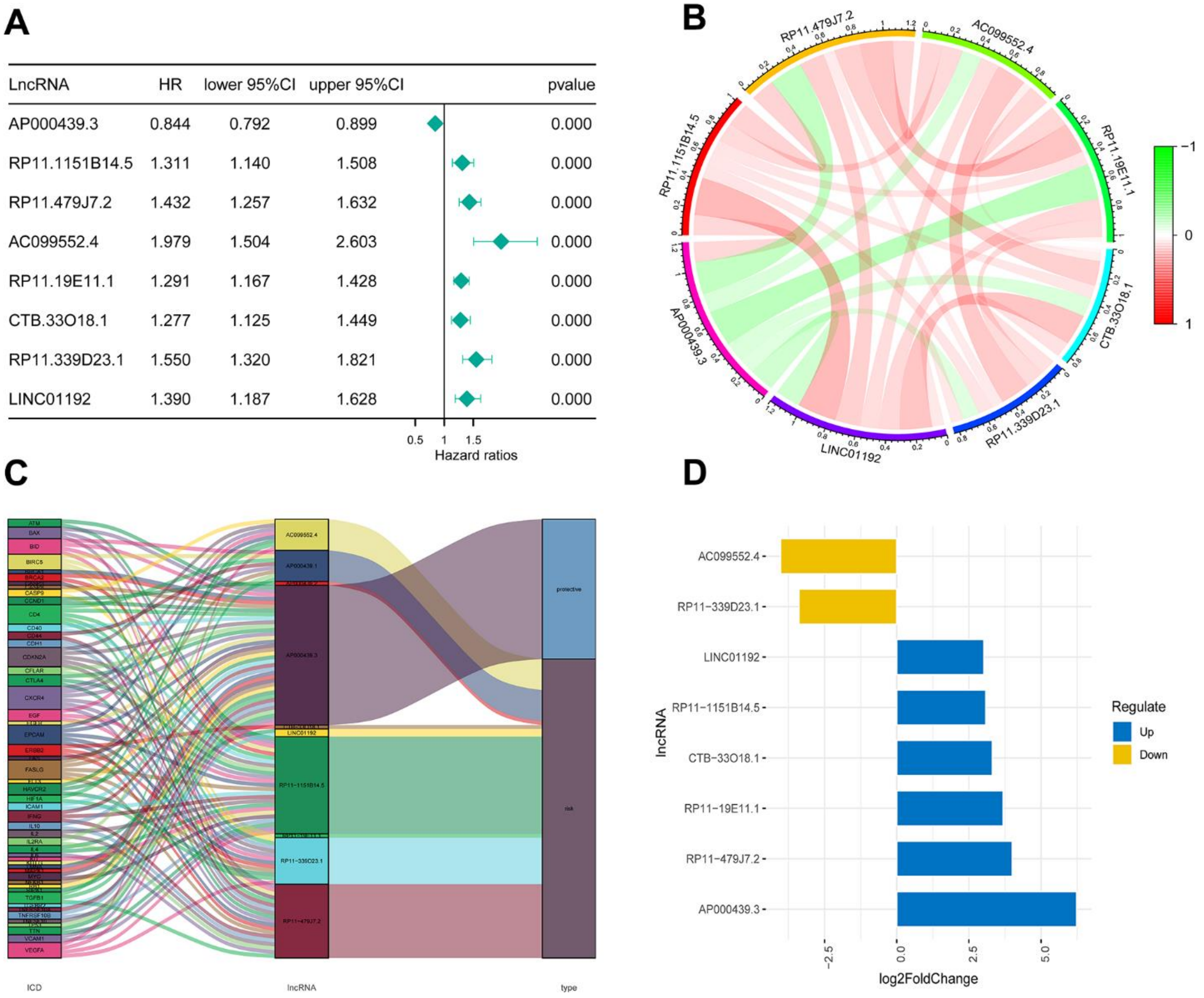

Supplementary Figure 1. Construct a prognostic model. Forest plots associated with multifactor regression (**A**); Correlation circle plot of 8 ICD-related lncRNAs (**B**); Mulberry plots of 8 ICD-related lncRNAs with ICD-related genes (**C**); Deviation plots indicating up- and down-regulation changes of 8 ICD-related lncRNAs (**D**).

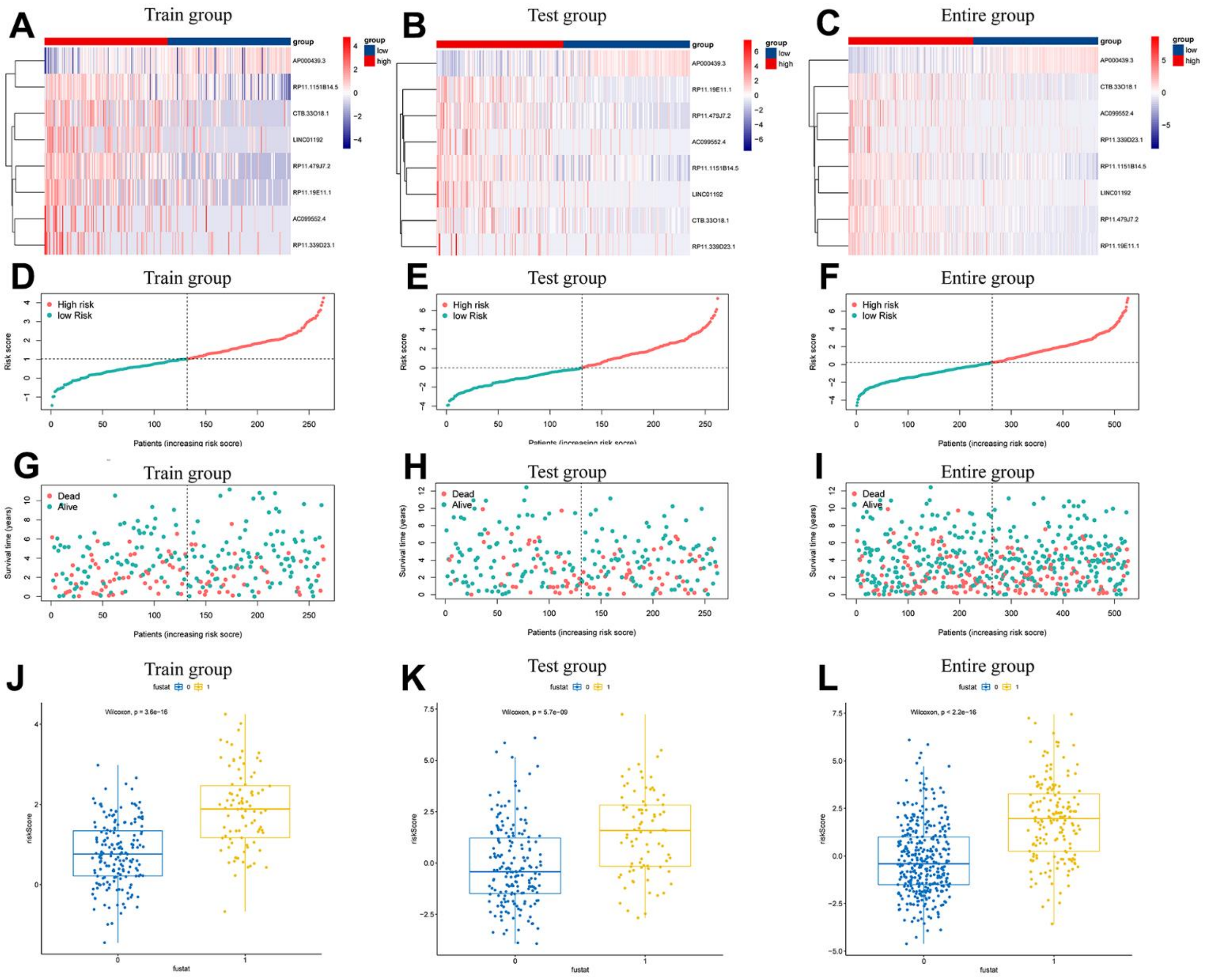

Supplementary Figure 2. The model prediction effect is validated by the training group, test group, and the entire group. Heat map of 8 ICD-related lncRNA expressions (**A**–**C**). Risk curve for risk scores (**D**–**F**) and Scatterplot (**G**–**I**) for the survival status of each patient; Scatterplot of risk scores of patients with different survival statuses (**J**–**L**).

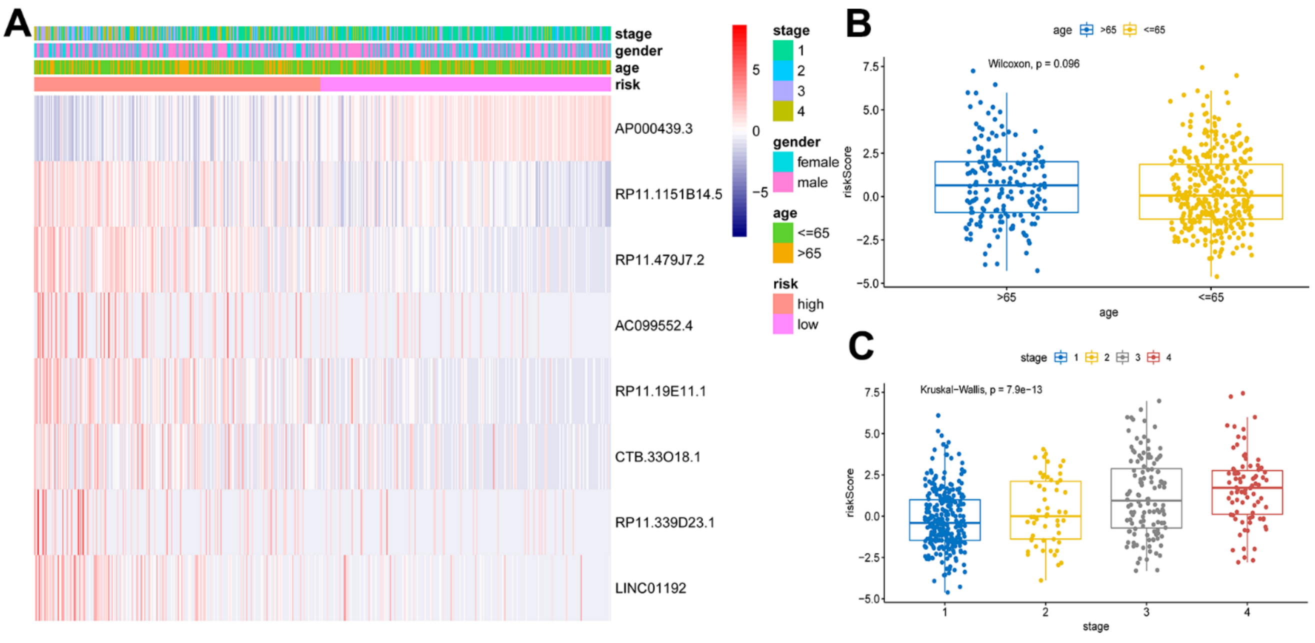

Supplementary Figure 3. Further validation of model effects. lncRNA signature expression heat map with clinical information (**A**); Correlation analysis of risk signature with age (**B**) and stage (**C**).

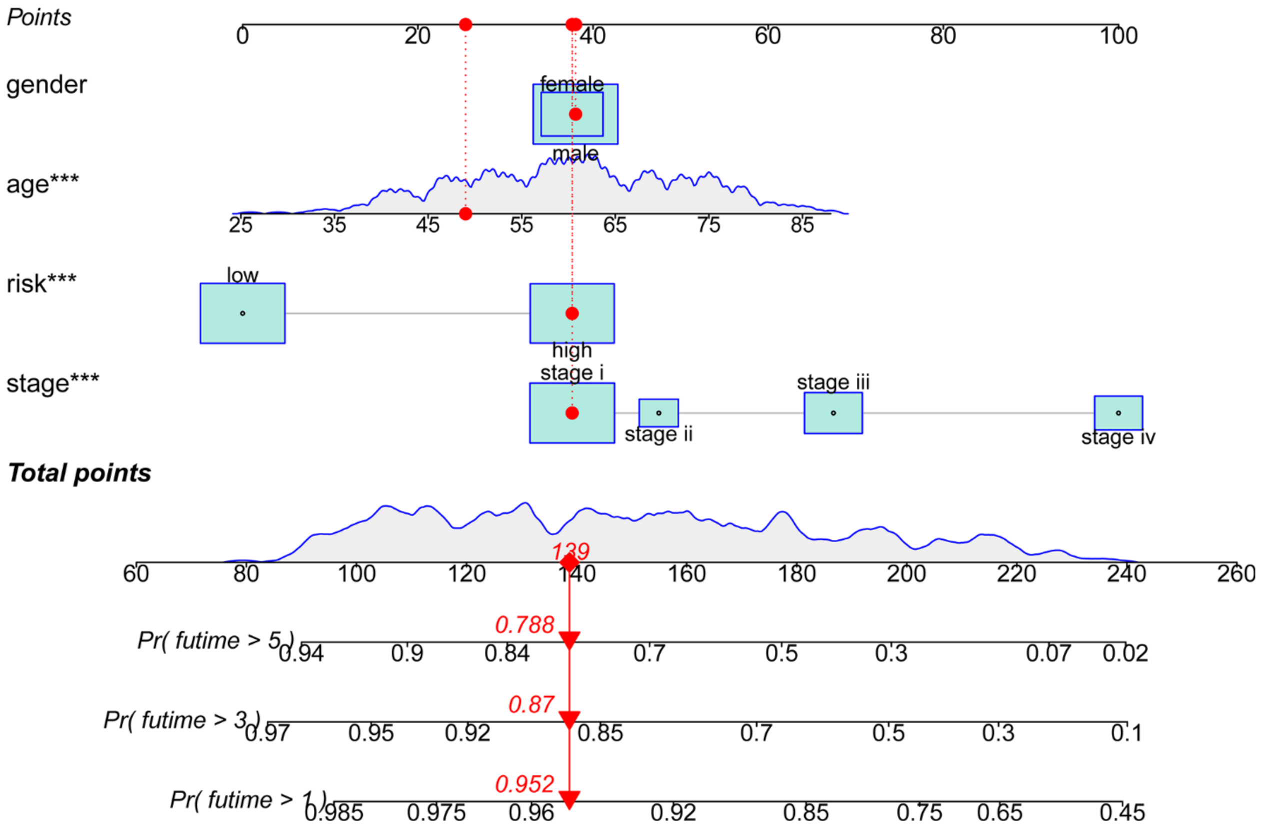

Supplementary Figure 4. Nomogram with patient verification.

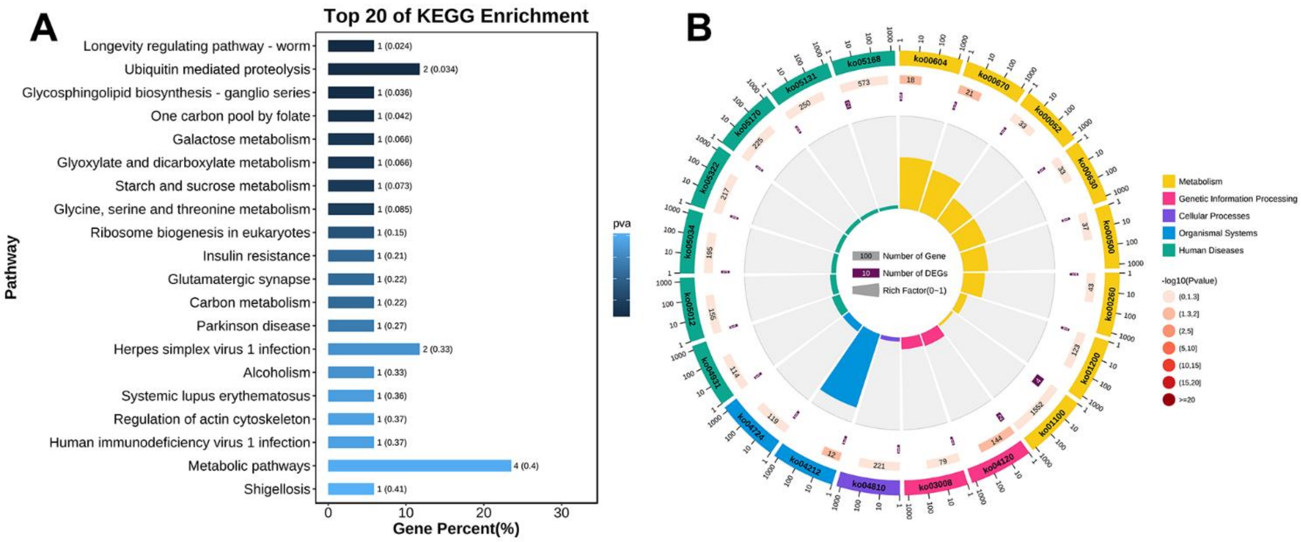

Supplementary Figure 5. KEGG enrichment analysis. Bar chart (**A**); Circle chart (**B**).

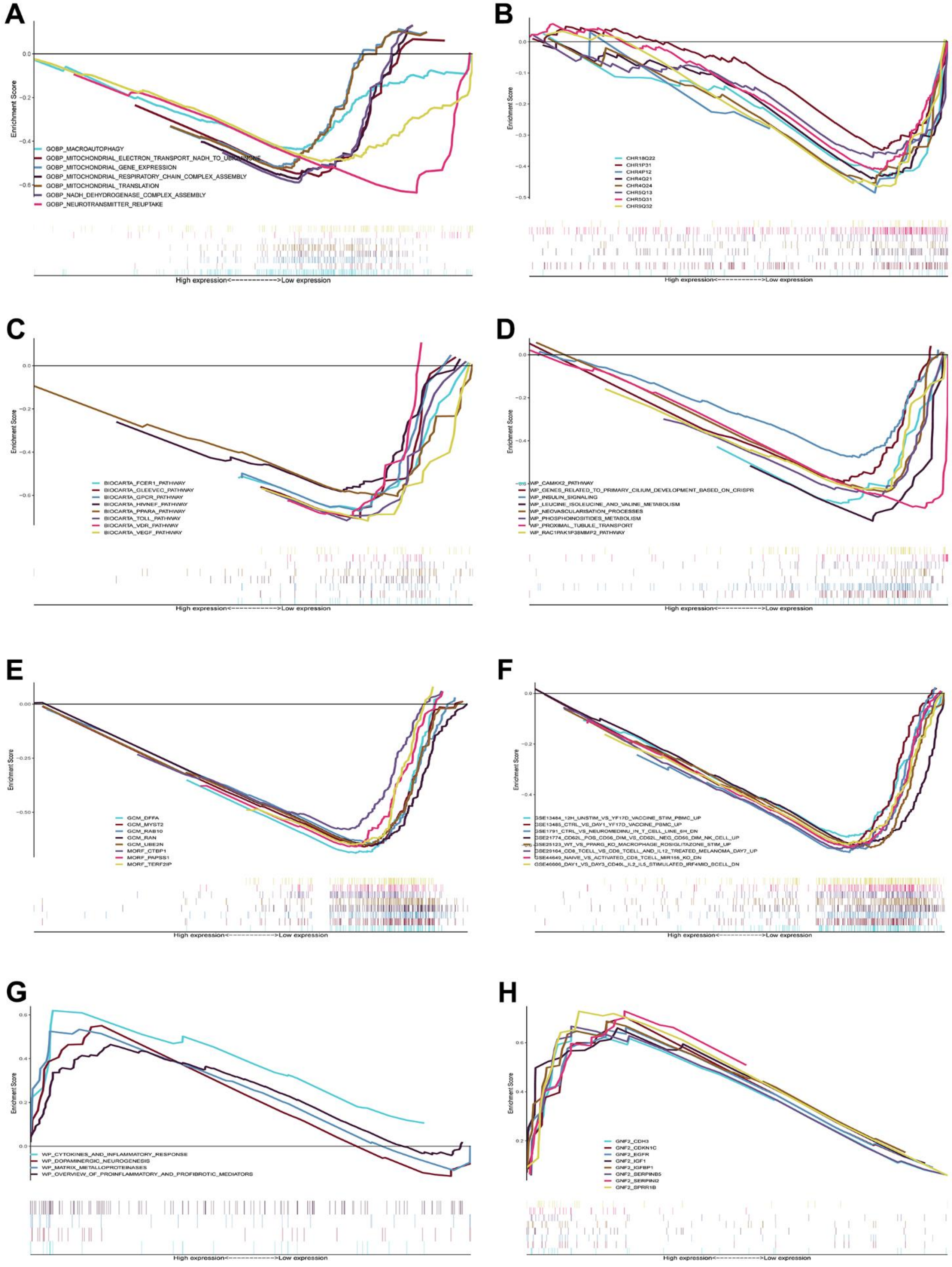

Supplementary Figure 6. GSEA enrichment analysis. Enrichment pathways in low-risk group in different gene sets (**A**–**F**); Enrichment pathways in high-risk group in different gene sets (**G**, **H**).

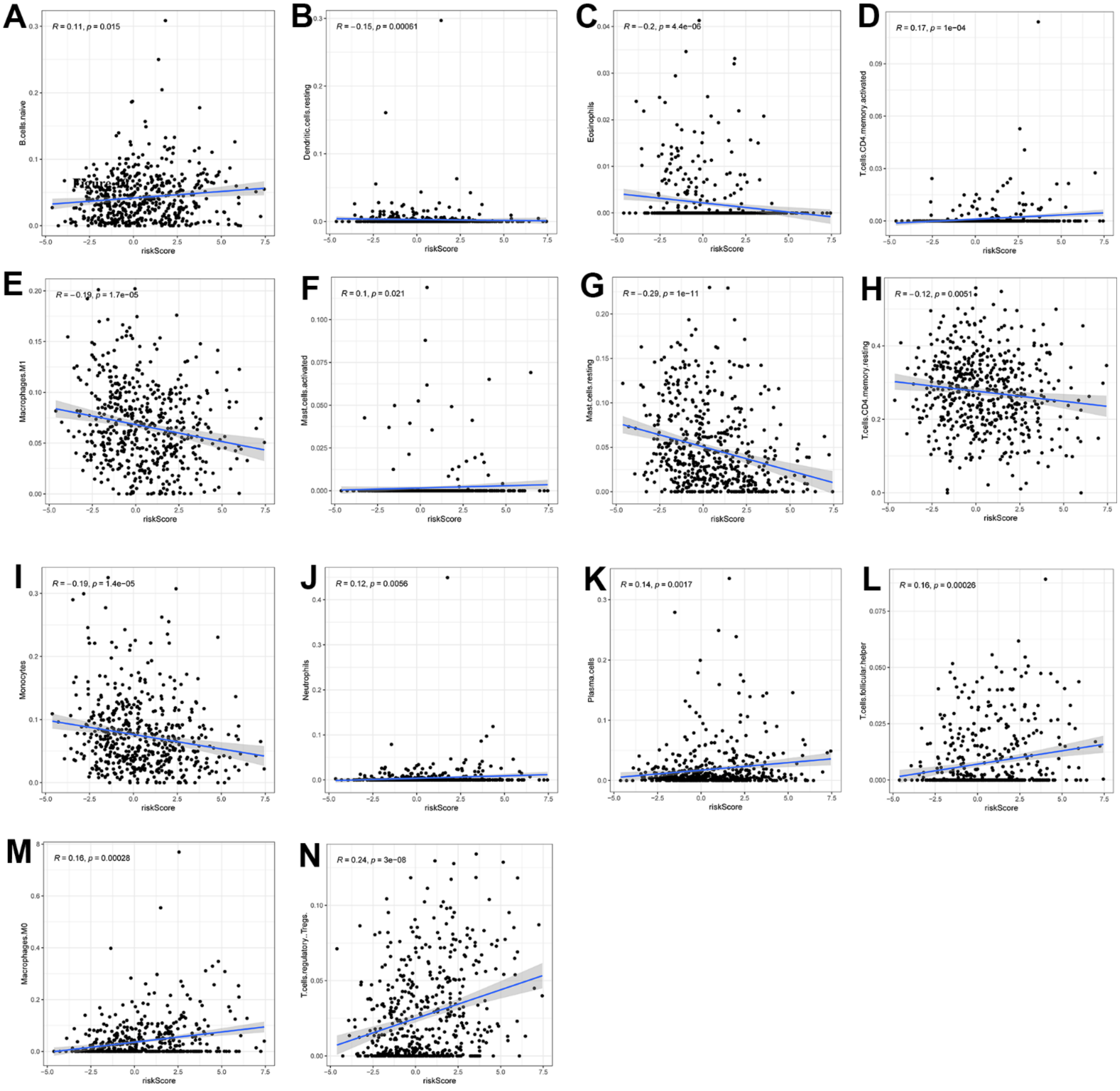

Supplementary Figure 7. Correlation plot of risk scores with immune cells. Scatter plot of the correlation between risk scores and naïve B cells (**A**), resting dendritic cells (**B**), eosinophils (**C**), activated CD4 memory T cells (**D**), macrophages M1 (**E**), activated mast cells (**F**), resting mast cells (**G**), resting CD4 memory T cells (**H**), monocytes (**I**), neutrophils (**J**), plasma cells (**K**), T follicular helper cells (**L**), macrophages M0 (**M**), regulatory T cells (Treg) (**N**).

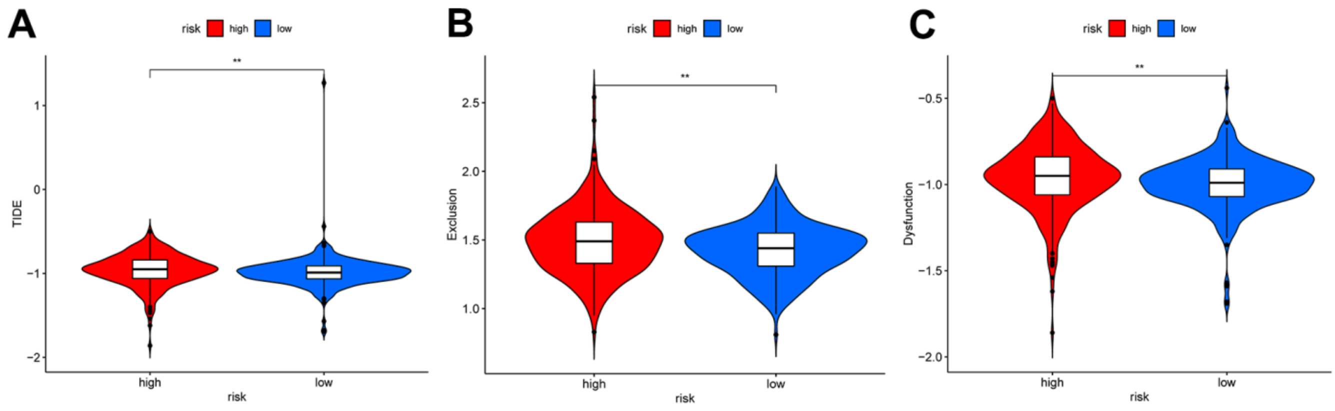

Supplementary Figure 8. The ICD-relevant lncRNA risk pattern in tumor therapy. Differences in TIDE (**A**) and immune exclusion (**B**) and dysfunction (**C**) in high and low-risk groups. *p < 0.05, **p < 0.01, ***p < 0.001.

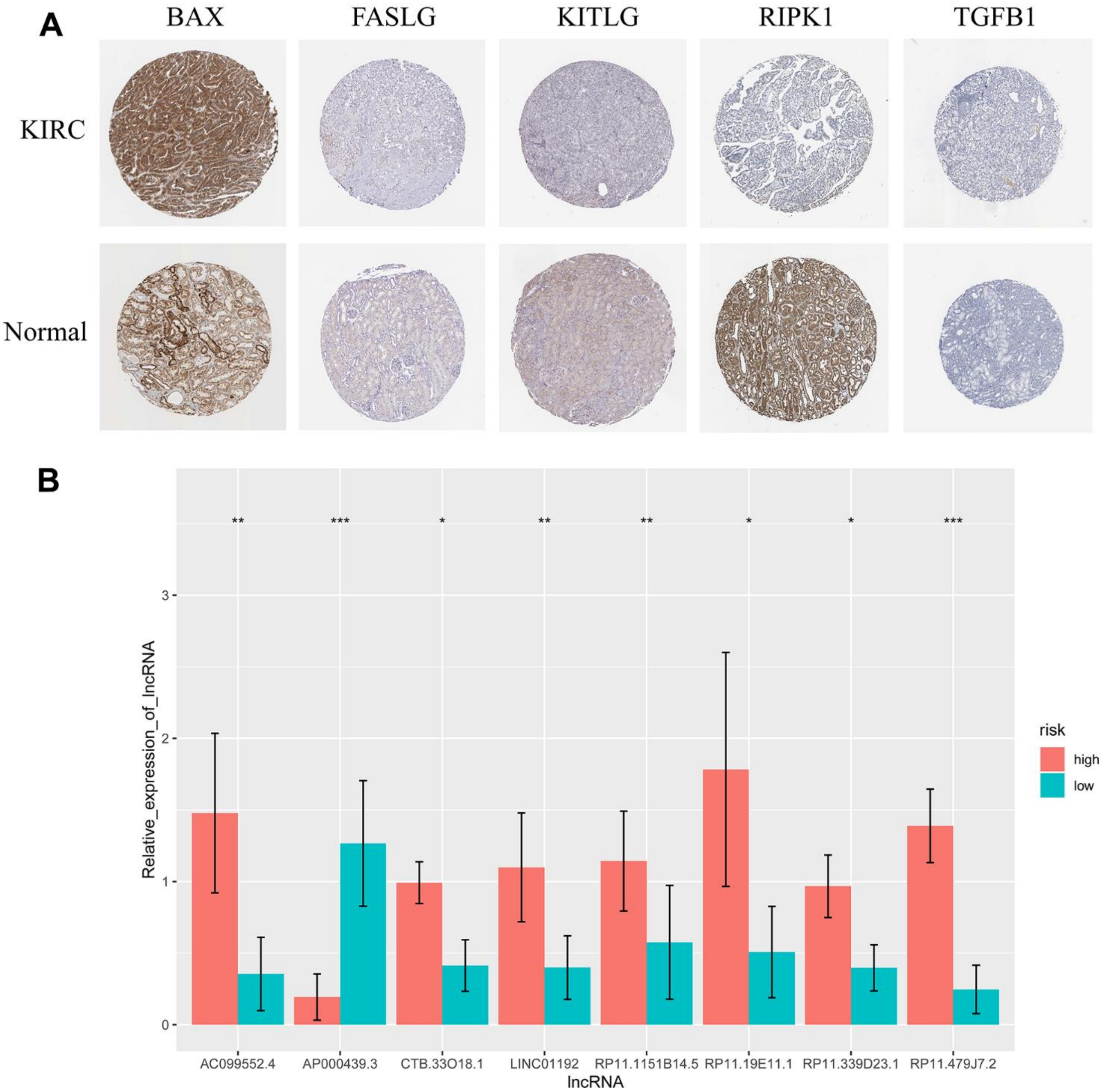

Supplementary Figure 9. *In vitro* experimental validation of the risk model. Immunohistochemical staining images of partial ICD-associated gene proteins in KIRC tissue and normal tissue (**A**); Relative expression of 8 ICD-related lncRNAs in different risk subgroups (**B**). *p < 0.05, **p < 0.01, ***p < 0.001, ****p < 0.0001.
