# Supplementary material for: Construction and validation of a model based on immunogenic cell death-associated lncRNAs to predict prognosis and direct therapy for kidney renal clear cell carcinoma
Source: Aging (Albany NY). 2023 Jun 27;15(12):5304–38. doi: 10.18632/aging.204741 (PMC10333057; doi:10.18632/aging.204741)
Supplement: Supplementary Tables [file aging-15-204741-s002.pdf]

SUPPLEMENTARY TABLES

Supplementary Table 1. Primer sequences for 8 ICD-related lncRNAs.

Gene id	Primer F	Primer R
AP000439.3	GTTTCTGGGCCCCTCCTTTG	GCTCCACACCTTTTTGCAGG
RP11.1151B14.5	AGAAAGCTGTGCAGTCTAAAGT	ACATACTCTTTCCAGGAGATGTAAT
RP11.479J7.2	TGGAACAAACTCCTAGTTTCTGGG	AGCCTGTGCTCCTAACCCTA
AC099552.4	GCCGACTTGGGGAAAGTTGA	CTCGGCTTGCTCCTCACAG
RP11.19E11.1	CAGAGTTTCAACGGCTCTGG	CATTACACAGGGAGAGCCAGG
CTB.33O18.1	GCCCCTGTTGTGGCTCATTG	TGGAGAGATTTGTGTTGAGAATGGA
RP11.339D23.1	TGACCAAATTCTGTTGGTACCTT	ACCACCAATTTTCCCCATTCTC
LINC01192	GCTGCATGCGTGGAAATGTT	GCTTTTGCACCGCTTCTGAC

Abbreviation: ICD, Immunogenic cell death.

Supplementary Table 2. 180 differentially expressed ICD-related lncRNAs.

LncRNA	HR (95%CI)	P-value	LncRNA	HR (95%CI)	P-value
RP5.1018K9.1	0.77(0.63-0.95)	0.02	RP11.457K10.1	1.36(1.06-1.75)	0.02
RP11.14C10.5	0.88(0.82-0.95)	0.00	RP11.622A1.2	0.82(0.71-0.95)	0.01
RP11.380J14.1	1.10(1.01-1.20)	0.03	IL21.AS1	1.11(1.01-1.21)	0.02
AP000696.2	1.23(1.05-1.44)	0.01	RP11.142M10.2	1.32(1.14-1.52)	0.00
CTD.2015G9.2	0.90(0.83-0.97)	0.01	RP11.120K18.2	1.28(1.10-1.50)	0.00
AP000439.3	0.84(0.79-0.90)	0.00	RP3.340N1.2	1.18(1.08-1.29)	0.00
LINC01020	0.77(0.64-0.93)	0.01	AC067959.1	0.84(0.78-0.91)	0.00
RP11.89B16.1	1.25(1.04-1.51)	0.02	RP11.400N13.3	1.15(1.05-1.26)	0.00
LUCAT1	1.16(1.04-1.28)	0.01	DKFZp434J0226	1.19(1.07-1.31)	0.00
CTD.2026K11.6	1.23(1.07-1.41)	0.00	RP11.206M11.7	1.14(1.05-1.24)	0.00
RP11.389O22.1	1.20(1.01-1.43)	0.03	CTD.2004A9.1	0.83(0.75-0.93)	0.00
AC073115.6	0.89(0.81-0.98)	0.01	CTA.392C11.1	1.23(1.10-1.39)	0.00
AC133644.2	1.58(1.29-1.94)	0.00	LINC00461	1.21(1.07-1.37)	0.00
LINC00299	1.20(1.04-1.38)	0.01	AC002331.1	1.37(1.18-1.60)	0.00
CTC.327F10.5	1.09(1.01-1.17)	0.03	RP11.567M16.2	1.49(1.19-1.86)	0.00
GATM.AS1	0.86(0.77-0.97)	0.01	RP13.259N13.2	1.85(1.20-2.85)	0.01
CTD.2020K17.1	1.24(1.09-1.41)	0.00	GS1.600G8.5	1.14(1.03-1.25)	0.01
MIAT	1.22(1.10-1.36)	0.00	RP11.617F23.2	1.23(1.08-1.41)	0.00
RP11.211G23.2	1.09(1.01-1.19)	0.04	FLJ22763	0.86(0.78-0.94)	0.00
LINC00944	1.20(1.05-1.36)	0.01	AP004372.1	1.26(1.14-1.39)	0.00
LINC00551	0.87(0.76-0.99)	0.04	RP11.429B14.4	1.20(1.08-1.33)	0.00
RP11.309M7.1	1.21(1.05-1.39)	0.01	KB.1043D8.8	0.76(0.63-0.92)	0.00
RP11.341G23.4	1.18(1.03-1.34)	0.01	RP11.145A3.1	1.21(1.06-1.38)	0.01
CTD.2131I18.1	1.41(1.01-1.98)	0.05	AF064858.7	1.19(1.03-1.38)	0.02
MIR155HG	1.23(1.07-1.41)	0.00	RP11.479J7.2	1.43(1.26-1.63)	0.00
LINC00443	0.75(0.59-0.94)	0.01	RP11.100G15.10	1.23(1.05-1.44)	0.01
AC012123.1	1.23(1.04-1.46)	0.02	RP11.586K2.1	1.15(1.07-1.24)	0.00
RP11.496I9.1	1.11(1.00-1.24)	0.04	PCSK6.AS1	1.18(1.03-1.35)	0.02
LINC00943	1.19(1.04-1.37)	0.01	RP11.804N13.1	1.22(1.09-1.36)	0.00
F11.AS1	0.87(0.78-0.98)	0.02	RP11.129I19.2	1.68(1.03-2.74)	0.04
RP11.361L15.4	0.86(0.79-0.95)	0.00	RP11.631F7.1	1.14(1.03-1.27)	0.01
AC015977.6	0.84(0.76-0.93)	0.00	AP000233.4	1.16(1.01-1.33)	0.04
RP11.167N4.2	1.11(1.01-1.22)	0.03	LINC00955	0.83(0.73-0.94)	0.00
LINC01428	0.91(0.83-0.99)	0.03	AC006262.5	1.24(1.15-1.34)	0.00
HNF4A.AS1	0.81(0.71-0.94)	0.00	AC099552.4	1.98(1.50-2.60)	0.00
LINC00158	1.16(1.03-1.31)	0.02	LINC01501	1.23(1.02-1.47)	0.03
RP11.44K6.4	1.14(1.01-1.29)	0.04	RP11.146E13.4	1.22(1.05-1.42)	0.01
ELDR	1.16(1.05-1.29)	0.00	LINC01281	1.22(1.07-1.39)	0.00
RP3.393E18.2	1.24(1.08-1.43)	0.00	RP11.815M8.1	1.21(1.09-1.34)	0.00
LINC00460	1.17(1.09-1.26)	0.00	LINC01583	1.18(1.06-1.33)	0.00
LINC01234	1.19(1.12-1.27)	0.00	CTD.2562J17.2	1.20(1.06-1.36)	0.00
RP11.417E7.1	1.29(1.13-1.46)	0.00	LINC00410	1.41(1.12-1.79)	0.00
RP11.255G12.3	0.81(0.71-0.92)	0.00	RP11.493L12.5	1.23(1.08-1.40)	0.00
RP11.1018N14.5	0.86(0.76-0.99)	0.03	RP11.407A16.3	1.18(1.03-1.34)	0.02
RP6.91H8.3	1.23(1.08-1.41)	0.00	LINC00705	1.25(1.03-1.51)	0.02
PROX1.AS1	1.37(1.15-1.61)	0.00	IL20RB.AS1	1.24(1.10-1.39)	0.00
RP11.475O23.2	0.75(0.61-0.91)	0.00	CTD.2532K18.2	1.19(1.08-1.31)	0.00
RP11.465L10.10	1.28(1.09-1.50)	0.00	RP5.984P4.6	1.14(1.02-1.27)	0.02

RP5.884M6.1	1.13(1.06-1.20)	0.00	AP000439.1	1.17(1.02-1.35)	0.03
RP11.291B21.2	1.25(1.09-1.42)	0.00	MYCNUT	1.34(1.00-1.78)	0.05
LINC00845	0.70(0.58-0.84)	0.00	AC008088.4	1.36(1.20-1.54)	0.00
CTD.2616J11.3	1.22(1.04-1.43)	0.01	AC011752.1	0.78(0.66-0.92)	0.00
CTD.2171N6.1	1.24(1.10-1.39)	0.00	RP11.275I4.2	1.22(1.02-1.45)	0.03
RP5.1172A22.1	1.12(1.03-1.23)	0.01	CTD.2527I21.15	1.17(1.04-1.31)	0.01
RP11.674P19.2	1.34(1.16-1.55)	0.00	AC116614.1	1.17(1.08-1.26)	0.00
RP11.395B7.2	1.21(1.07-1.36)	0.00	RP11.344P13.4	1.36(1.16-1.60)	0.00
AC068492.1	1.27(1.10-1.48)	0.00	RP11.543G18.1	1.34(1.09-1.64)	0.01
TMEM92.AS1	1.25(1.10-1.42)	0.00	RP11.486M23.2	1.21(1.04-1.41)	0.01
RP11.568J23.8	1.13(1.01-1.27)	0.03	RP11.356I2.1	1.44(1.21-1.71)	0.00
RP11.556E13.1	1.18(1.01-1.38)	0.03	RP11.73M14.1	1.25(1.09-1.44)	0.00
RP11.1151B14.5	1.31(1.14-1.51)	0.00	RP11.909N17.2	1.19(1.08-1.31)	0.00
AFAP1.AS1	1.11(1.02-1.21)	0.02	LL22NC03.63E9.3	1.21(1.05-1.38)	0.01
LINC00704	1.24(1.09-1.42)	0.00	LINC01411	1.10(1.02-1.18)	0.01
RP11.551L14.4	1.22(1.08-1.38)	0.00	RP11.191N8.2	1.29(1.15-1.46)	0.00
LL22NC03.N14H11.1	1.22(1.07-1.40)	0.00	RP11.478J18.2	1.33(1.14-1.55)	0.00
RP11.879F14.1	0.80(0.65-0.98)	0.03	RP11.19E11.1	1.29(1.17-1.43)	0.00
RP11.414H23.3	1.14(1.01-1.28)	0.03	RP3.417L20.4	1.33(1.08-1.65)	0.01
RP5.988G17.1	0.79(0.66-0.95)	0.01	HOTTIP	1.25(1.12-1.40)	0.00
RP11.322D14.2	1.31(1.14-1.52)	0.00	CTC.241F20.4	1.14(1.01-1.29)	0.04
LINC00264	1.17(1.01-1.36)	0.04	NPSR1.AS1	1.16(1.02-1.33)	0.02
RP4.547N15.3	1.45(1.08-1.96)	0.01	RP11.377G16.2	1.13(1.00-1.27)	0.05
AP000697.6	1.79(1.39-2.30)	0.00	LINC01351	1.28(1.04-1.59)	0.02
CTD.2023M8.1	1.36(1.18-1.57)	0.00	RP11.429E11.2	1.21(1.07-1.37)	0.00
RP11.10J5.1	1.14(1.01-1.28)	0.04	RP11.440G9.1	1.29(1.16-1.44)	0.00
CTD.2357A8.3	1.41(1.24-1.6)	0.00	RP11.414H23.2	1.27(1.09-1.47)	0.00
EGLN3.AS1	0.84(0.72-0.98)	0.02	CTD.2008P7.8	1.23(1.02-1.49)	0.03
CTD.2553C6.1	1.20(1.04-1.37)	0.01	CTB.178M22.1	1.16(1.02-1.34)	0.03
RP11.366L20.2	1.15(1.02-1.30)	0.02	RP11.279O17.1	1.18(1.02-1.36)	0.02
RP11.84D1.1	1.24(1.05-1.46)	0.01	CTB.33O18.1	1.28(1.13-1.45)	0.00
AC010729.1	1.24(1.08-1.43)	0.00	WASF3.AS1	1.16(1.01-1.33)	0.03
LINC00380	0.67(0.49-0.91)	0.01	RP11.616M22.3	1.19(1.01-1.41)	0.04
RP3.380B4.1	0.78(0.62-0.99)	0.04	RP11.339D23.1	1.55(1.32-1.82)	0.00
RP11.70D24.4	1.17(1.02-1.36)	0.03	LINC01405	1.24(1.08-1.43)	0.00
AC006262.4	1.23(1.10-1.38)	0.00	LINC01551	1.18(1.05-1.34)	0.01
PTCSC3	0.88(0.78-0.99)	0.04	RP11.161D15.1	1.13(1.02-1.25)	0.01
RP11.108E14.1	0.88(0.78-0.99)	0.04	RP11.96B2.1	1.14(1.02-1.28)	0.02
RP11.184A2.2	0.79(0.66-0.96)	0.02	LINC01192	1.39(1.19-1.63)	0.00
RP1.142L7.9	1.42(1.24-1.62)	0.00	RP11.94A24.1	1.12(1.00-1.26)	0.05
RP11.14C10.3	1.15(1.00-1.33)	0.05	RP11.161D15.3	1.27(1.11-1.45)	0.00
RP1.105O18.1	0.83(0.71-0.96)	0.01	AC092484.1	1.29(1.13-1.46)	0.00

Abbreviation: ICD, Immunogenic cell death; HR, Hazard Ratio; CL, Confidence level.

Supplementary Table 3. 14 differentially expressed ICD-related lncRNAs.

LncRNA	Coef
AP000439.3	-0.05695
LINC01234	0.032357
RP11.1151B14.5	0.026259
CTD.2357A8.3	0.025506
RP1.142L7.9	0.039787
AP004372.1	0.008293
RP11.479J7.2	0.051337
AC006262.5	0.030434
AC099552.4	0.000912
AC008088.4	0.034797
RP11.19E11.1	0.01574
CTB.33O18.1	0.004644
RP11.339D23.1	0.121664
LINC01192	0.007471

Abbreviation: ICD, Immunogenic cell death.

Supplementary Table 4. GSEA pathways for different risk groups.

Pathways	Group
**C1.all.v2022.1.Hs.symbols.gmt**	
CHR18Q22	Low risk
CHR1P31	Low risk
CHR4P12	Low risk
CHR4Q21	Low risk
CHR4Q24	Low risk
CHR5Q13	Low risk
CHR5Q31	Low risk
CHR9Q32	Low risk
**C2.cp.wikipathways.v2022.1.Hs.symbols.gmt**	
WP_GENES_RELATED_TO_PRIMARY_CILIUM_DEVELOPMENT_BASED_ON_CRISPR	Low risk
WP_INSULIN_SIGNALING	Low risk
WP_LEUCINE_ISOLEUCINE_AND_VALINE_METABOLISM	Low risk
WP_NEOVASCULARISATION_PROCESSES	Low risk
WP_PHOSPHOINOSITIDES_METABOLISM	Low risk
WP_PROXIMAL_TUBULE_TRANSPORT	Low risk
WP_RAC1PAK1P38MMP2_PATHWAY	Low risk
WP_CAMKK2_PATHWAY	Low risk
WP_CYTOKINES_AND_INFLAMMATORY_RESPONSE	High risk
WP_DOPAMINERGIC_NEUROGENESIS	High risk
WP_MATRIX_METALLOPROTEINASES	High risk
WP_OVERVIEW_OF_PROINFLAMMATORY_AND_PROFIBROTIC_MEDIATORS	High risk
**C4.cgn.v2022.1.Hs.symbols.gmt**	
GCM_DFFA	Low risk
GCM_MYST2	Low risk
GCM_RAB10	Low risk
GCM_RAN	Low risk
GCM_UBE2N	Low risk
MORF_CTBP1	Low risk
MORF_PAPSS1	Low risk
MORF_TERF2IP	Low risk
GNF2_CDH3	High risk
GNF2_CDKN1C	High risk
GNF2_EGFR	High risk
GNF2_IGF1	High risk
GNF2_IGFBP1	High risk
GNF2_SERPINB5	High risk
GNF2_SERPINI2	High risk
GNF2_SPRR1B	High risk
**C5.go.bp.v2022.1.Hs.symbols.gmt**	
GOBP_MACROAUTOPHAGY	Low risk
GOBP_MITOCHONDRIAL_ELECTRON_TRANSPORT_NADH_TO_UBIQUINONE	Low risk
GOBP_MITOCHONDRIAL_GENE_EXPRESSION	Low risk
GOBP_MITOCHONDRIAL_RESPIRATORY_CHAIN_COMPLEX_ASSEMBLY	Low risk
GOBP_MITOCHONDRIAL_TRANSLATION	Low risk
GOBP_NADH_DEHYDROGENASE_COMPLEX_ASSEMBLY	Low risk
GOBP_NEUROTRANSMITTER_REUPTAKE	Low risk
**C7.immunesigdb.v2022.1.Hs.symbols.gmt**	
GSE13484_12H_UNSTIM_VS_YF17D_VACCINE_STIM_PBMC_UP	Low risk
GSE13485_CTRL_VS_DAY1_YF17D_VACCINE_PBMC_UP	Low risk
GSE1791_CTRL_VS_NEUROMEDINU_IN_T_CELL_LINE_6H_DN	Low risk
GSE21774_CD62L_POS_CD56_DIM_VS_CD62L_NEG_CD56_DIM_NK_CELL_UP	Low risk

GSE25123_WT_VS_PPARG_KO_MACROPHAGE_ROSIGLITAZONE_STIM_UP	Low risk
GSE29164_CD8_TCELL_VS_CD8_TCELL_AND_IL12_TREATED_MELANOMA_DAY7_UP	Low risk
GSE44649_NAIVE_VS_ACTIVATED_CD8_TCELL_MIR155_KO_DN	Low risk
GSE46606_DAY1_VS_DAY3_CD40L_IL2_IL5_STIMULATED_IRF4MID_BCELL_DN	Low risk
**C2.cp.biocarta.v2022.1.Hs.symbols.gmt**	
BIOCARTA_FCER1_PATHWAY	Low risk
BIOCARTA_GLEEVEC_PATHWAY	Low risk
BIOCARTA_GPCR_PATHWAY	Low risk
BIOCARTA_HIVNEF_PATHWAY	Low risk
BIOCARTA_PPARA_PATHWAY	Low risk
BIOCARTA_TOLL_PATHWAY	Low risk
BIOCARTA_VDR_PATHWAY	Low risk
BIOCARTA_VEGF_PATHWAY	Low risk

Abbreviation: GSEA, Gene Set Enrichment Analysis.

Supplementary Table 5. Antineoplastic drug sensitivity information (sensitive group: low).

Target pathways	Low-risk group	High-risk group	P-value
	IC50 (25%-75%)	IC50 (25%-75%)	
**WNT signaling**			
WIKI4	42.29(37.88-47.68)	75.50(65.11-88.45)	0.00
VX.11e	16.56(14.04-20.06)	38.91(34.91-42.75)	0.01
**Unclassified**			
Sepantronium.bromide	0.01(0.01-0.02)	36.44(30.60-44.06)	0.00
Gallibiscoquinazole	12.86(11.58-14.66)	13.57(11.74-15.76)	0.05
ABT737	9.23(6.81-11.59)	129.26(95.99-168.98)	0.01
**RTK signaling**			
Sabutoclax	0.72(0.60-0.88)	13.38(11.02-15.53)	0.00
**Protein stability and degradation**			
NVP.ADW742	13.80(10.81-18.05)	89.56(70.55-116.29)	0.00
**Other, kinases**			
WEHI.539	34.60(28.26-41.18)	37.74(30.64-48.38)	0.00
PAK_5339	10.51(8.98-12.42)	23.54(18.01-32.21)	0.00
JAK_8517	19.33(15.09-25.41)	62.25(50.55-77.02)	0.00
Ipatasertib	33.70(26.00-43.77)	134.84(116.90-152.35)	0.00
IGF1R_3801	5.00(3.86-6.63)	95.09(70.00-138.36)	0.04
GSK343	16.56(14.35-19.60)	124.03(105.44-149.96)	0.00
AZD5153	5.25(4.39-6.93)	17.14(12.59-23.20)	0.00
AT13148	33.19(26.12-44.75)	199.26(167.97-245.92)	0.00
**Other**			
Zoledronate	40.71(34.13-49.19)	43.54(35.16-53.14)	0.00
LY2109761	153.27(126.84-189.48)	179.12(143.41-246.60)	0.00
Ibrutinib	78.65(62.87-98.99)	173.16(135.80-230.66)	0.01
CDK9_5576	0.64(0.54-0.84)	5.16(3.23-8.01)	0.00
**Mitosis**			
Eg5_9814	0.04(0.04-0.06)	0.08(0.06-0.15)	0.00
**Hormone-related**			
Teniposide	1.83(1.26-2.53)	33.56(29.52-38.86)	0.03
**Genome integrity**			
Telomerase.Inhibitor.IX	1.69(1.37-2.08)	22.22(15.6-34.44)	0.00
MK.8776	25.33(20.38-34.05)	68.51(51.11-93.33)	0.02
MIM1	49.25(41.24-59.66)	99.13(79.42-130.58)	0.00
**ERK MAPK signaling**			
ULK1_4989	12.24(9.56-16.2)	16.5(13.11-19.55)	0.02
Cediranib	7.35(6.24-9.64)	91.59(67.86-116.00)	0.00
**EGFR signaling**			
Ribociclib	43.98(39.57-51.04)	54.24(43.41-68.68)	0.03
GNE.317	1.73(1.48-1.98)	26.65(21.38-32.28)	0.00
Foretinib	2.56(2.16-3.17)	14.12(11.69-17.03)	0.00
AZD3759	13.51(11.58-16.15)	15.38(12.67-18.39)	0.00
**DNA replication**			
Topotecan	1.27(0.95-1.77)	1.40(0.86-2.19)	0.00
Mitoxantrone	1.98(1.35-2.79)	421.96(336.39-553.11)	0.00
KRAS..G12C..Inhibitor.12	80.02(63.25-100.65)	136.39(117.1-163.16)	0.03
CDK9_5038	0.11(0.08-0.14)	20.33(14.16-30.94)	0.00
**Chromatin other**			
ERK_6604	33.68(28.64-39.57)	160.16(129.28-215.77)	0.00

Vinorelbine	0.05(0.03-0.07)	3.90(3.29-5.00)	0.00
**Cell cycle**			
PRT062607	25.53(21.00-32.87)	41.85(37.07-49.75)	0.02
Osimertinib	4.75(3.67-6.60)	33.40(23.53-50.41)	0.02
**Apoptosis regulation**			
MG.132	0.20(0.19-0.22)	45.92(37.49-57.63)	0.00
JQ1	14.16(12.75-16.31)	143.92(115.17-175.26)	0.00
**Alkylating Agents**			
BPD.00008900	86.84(71.70-105.46)	446.46(376.50-555.49)	0.00

Abbreviation: IC50, half maximal inhibitory concentration.

Supplementary Table 6. Antineoplastic drug sensitivity information (sensitive group: high).

Target pathway	Low-risk group	High-risk group	P-value
	IC50 (25%-75%)	IC50 (25%-75%)	
**WNT signaling**			
AGI.6780	61.49(54.08-70.18)	11.39(9.98-12.99)	0.00
**Unclassified**			
Elephantin	35.7(29.36-41.13)	27.88(23.13-36.02)	0.00
**RTK signaling**			
Savolitinib	14.35(12.02-16.16)	11.23(7.64-14.67)	0.00
Oxaliplatin	158.03(120.85-197.77)	60.21(40.16-86.14)	0.01
Carmustine	424.01(361.17-492.53)	8.56(6.45-12.26)	0.03
**Protein stability and degradation**			
LJI308	159.58(139.34-193.48)	0.09(0.06-0.14)	0.01
BIBR.1532	140.36(118.87-165.55)	0.01(0.01-0.01)	0.01
**PI3K/MTOR signaling**			
Uprosertib	16.82(12.41-22.07)	14.23(10.67-20.88)	0.00
Podophyllotoxin.bromide	0.51(0.42-0.64)	0.11(0.08-0.15)	0.00
BDP.00009066	10.18(8.8-11.93)	0.70(0.59-0.85)	0.00
AZD6482	24.15(21.27-28.18)	22.25(19.19-26.01)	0.00
AZ6102	10.71(9.72-11.95)	7.40(6.65-8.46)	0.00
AGI.5198	111.82(99.84-125.75)	38.22(28-52.07)	0.00
Afuresertib	12.82(10.3-16.04)	11.55(9.15-15.33)	0.00
**Other, kinases**			
UMI.77	14.45(12.16-17.16)	7.78(5.57-10.70)	0.01
Sinularin	32.78(29.27-38.27)	13.17(10.80-16.78)	0.00
AMG.319	123.96(101.37-152.52)	6.80(4.69-9.84)	0.00
**Other**			
VSP34_8731	11.11(9.72-13.21)	9.78(7.93-11.96)	0.01
TAF1_5496	50.63(41.84-59.02)	46.21(36.06-57.84)	0.00
P22077	80.11(65.41-97.91)	1.72(1.23-2.41)	0.00
Dactinomycin	0.09(0.07-0.11)	0.08(0.06-0.10)	0.00
**Mitosis**			
Vincristine	0.17(0.11-0.26)	0.04(0.02-0.06)	0.00
Venetoclax	8.66(7.40-9.96)	0.14(0.09-0.25)	0.01
VE821	57.31(43.43-82.9)	0.02(0.01-0.03)	0.00
OF.1	59.52(53.31-67.57)	0.05(0.04-0.08)	0.00
Docetaxel	0.09(0.07-0.14)	0.01(0.01-0.01)	0.00
**Metabolism**			

GSK591	96.54(83.48-116.68)	42.8(32.43-57)	0.01
Dihydrorotenone	2.49(2.14-2.77)	0.01(0.01-0.02)	0.00
**Hormone-related**			
GDC0810	140.88(124.09-155.74)	131.69(114.65-156.33)	0.00
Fulvestrant	92.37(80.02-110.15)	17.73(14.89-20.70)	0.00
**Genome integrity**			
AZD6738	8.40(6.57-10.84)	6.60(4.60-9.41)	0.05
**ERK MAPK signaling**			
Ulixertinib	9.07(7.60-10.72)	1.90(1.38-2.62)	0.00
Selumetinib	63.91(51.39-79.99)	13.68(13.06-14.24)	0.00
OTX015	11.46(9.38-15.25)	1.66(1.31-2.27)	0.00
Fludarabine	153.12(122.24-196.14)	28.46(21.96-37.26)	0.03
**EGFR signaling**			
ML323	84.62(74.47-99.24)	6.00(4.16-8.62)	0.03
JAK1_8709	65.14(56.32-76.69)	18.54(15.03-22.95)	0.00
Acetalax	139.44(109.12-176.62)	6.46(4.90-8.35)	0.01
**DNA replication**			
Ulixertinib	14.8(12.71-17.91)	0.91(0.61-1.29)	0.00
Picolinici.acid	166.22(146.25-191.24)	26.33(19.64-34.06)	0.03
Nelarabine	385.41(321.70-485.20)	143.42(108.65-188.38)	0.01
MIRA.1	228.08(179.69-298.01)	1.64(1.04-2.56)	0.00
IRAK4_4710	144.97(131.83-158.61)	11.42(7.24-17.22)	0.00
GSK2578215A	144.31(122.45-167.17)	0.41(0.21-0.67)	0.00
ERK_2440	14.53(12.50-17.10)	0.31(0.22-0.42)	0.00
BMS.754807	1.32(0.82-2.32)	0.08(0.05-0.11)	0.00
**Cytoskeleton**			
I.BRD9	75.04(59.99-99.81)	16.04(12.94-20.44)	0.00
**Chromatin other**			
MN.64	112.51(96.58-130.52)	10.54(8.29-15.62)	0.00
AZD4547	17.18(14.60-20.68)	4.81(3.78-6.78)	0.00
Entospletinib	39.20(33.69-45.94)	8.07(6.12-11.3)	0.01
**Cell cycle**			
Buparlisib	2.44(2.19-2.88)	0.08(0.06-0.12)	0.00
AZD8186	24.1(20.19-29.98)	0.91(0.67-1.33)	0.00
AZD5363	18.99(15.07-23.95)	8.15(6.38-10.41)	0.00
**Apoptosis regulation**			
Pyridostatin	30.02(24.78-38.68)	0.59(0.49-0.77)	0.01
AZD5991	72.74(52.64-102.08)	7.57(4.54-14.02)	0.01

Abbreviation: IC50, half maximal inhibitory concentration.

Supplementary Table 7. Antineoplastic drug sensitivity information (no obviously sensitive group).

Target pathway	Low-risk group	High-risk group	P-value
	IC50 (25%-75%)	IC50 (25%-75%)	
**WNT signaling**			
SB216763	179.83(156.6-212.83)	181.75(148.47-225.68)	0.59
LGK974	55.86(48.16-64.32)	54.87(44.85-66.89)	0.43
**Unclassified**			
Dihydrorotenone	2.49(2.14-2.77)	2.46(2.11-2.94)	0.61
**RTK signaling**			
Axitinib	20.05(16.51-25.95)	19.45(15.25-26.63)	0.24
Staurosporine	0.04(0.04-0.06)	0.04(0.03-0.06)	0.10
Crizotinib	23.12(18.48-30.73)	24.38(17.58-34.72)	0.34
AZD1332	45.62(39.19-55.18)	44.39(34.49-56.93)	0.11
AZD4547	17.18(14.6-20.68)	17.49(14.11-22.72)	0.55
Foretinib	2.56(2.16-3.17)	2.42(1.96-3.27)	0.07
**Protein stability and degradation**			
ML323	84.62(74.47-99.24)	87.29(72.25-107.26)	0.21
**PI3K/MTOR signaling**			
Dactolisib	0.19(0.16-0.23)	0.18(0.15-0.23)	0.10
Pictilisib	3.91(3.17-4.92)	3.78(2.97-5.04)	0.75
Uprosertib	18.46(16.09-23.5)	19.21(15.26-25.18)	0.92
Alpelisib	33.17(22.44-51.15)	33.6(22.19-51.25)	0.75
Taselisib	7.13(4.36-11.73)	8.11(4.65-13.63)	0.09
CZC24832	156.48(134.35-191.13)	153.06(119.65-191.94)	0.15
Buparlisib	2.44(2.19-2.88)	2.53(2.14-2.94)	0.53
AZD8186	24.1(20.19-29.98)	25.55(19.76-34.36)	0.06
Ipatasertib	33.70(26.00-43.77)	32.39(24.54-42.96)	0.29
LJI308	159.58(139.34-193.48)	155.09(124.33-192.56)	0.06
**Other, kinases**			
Dasatinib	5.15(4.95-5.39)	5.08(4.83-5.42)	0.05
Ruxolitinib	118.28(96.65-149.94)	125.76(97.35-165.38)	0.08
Entospletinib	39.2(33.69-45.94)	39.71(31.21-51.33)	0.95
JAK_8517	19.33(15.09-25.41)	18.41(12.84-29.02)	0.22
**Other**			
Eg5_9814	0.04(0.04-0.06)	0.04(0.03-0.06)	0.11
**Mitosis**			
ZM447439	17.85(15.07-21.97)	17.53(13.74-23.70)	0.31
Alisertib	6.51(4.69-9.11)	6.03(3.85-10.49)	0.23
Tozasertib	17.53(13.45-21.76)	17.7(13.09-24.93)	0.32
**JNK and p38 signaling**			
Doramapimod	86.53(79.92-96.86)	86.61(77.31-98.81)	0.89
**IGF1R signaling**			
GSK1904529A	74.93(60.29-91.33)	72.98(58.94-90.4)	0.73
Linsitinib	43.21(33.21-55.65)	41.31(32.23-58.00)	0.63
IGF1R_3801	5.00(3.86-6.63)	4.86(3.38-7.29)	0.39
**Hormone-related**			
Fulvestrant	92.37(80.02-110.15)	90.44(74.35-110.84)	0.17
**Genome integrity**			
NU7441	13.06(11.89-14.56)	13.07(11.46-15.45)	0.54
Niraparib	73.26(59.10-96.31)	73.10(49.36-98.28)	0.13

VE821	57.31(43.43-82.9)	55.63(37.10-83.44)	0.27
**ERK MAPK signaling**			
ERK_2440	14.53(12.50-17.10)	14.09(10.91-17.32)	0.07
Selumetinib	63.91(51.39-79.99)	67.34(45.39-89.41)	0.56
Ulixertinib	9.07(7.60-10.72)	8.88(7.46-10.70)	0.60
**DNA replication**			
Oxaliplatin	42.82(31.71-54.63)	41.10(29.22-58.18)	0.59
Temozolomide	394.3(318.49-486.01)	378.71(296.19-510.18)	0.23
Cyclophosphamide	172.72(151.42-208.87)	165.82(138.41-204.14)	0.06
Fludarabine	153.12(122.24-196.14)	148.68(109.94-205.26)	0.46
**Cytoskeleton**			
PAK_5339	10.51(8.98-12.42)	10.73(8.89-13.17)	0.31
**Chromatin other**			
PFI3	184.47(161.04-216.21)	188.11(157.32-226.18)	0.59
EPZ5676	241.95(203.57-297.67)	258.07(205.18-319.53)	0.18
GSK343	16.56(14.35-19.60)	16.15(13.37-19.58)	0.14
GSK591	96.54(83.48-116.68)	90.85(75.51-116.73)	0.12
**Cell cycle**			
Dinaciclib	0.06(0.05-0.07)	0.06(0.04-0.07)	0.07
CDK9_5576	0.64(0.54-0.84)	0.62(0.49-0.82)	0.13
**Apoptosis regulation**			
Navitoclax	6.45(4.52-9.48)	6.70(4.62-10.13)	0.57
AZD5991	72.74(52.64-102.08)	72.66(47.73-113.83)	0.82
Venetoclax	8.66(7.40-9.96)	8.47(6.90-11.01)	0.83
ABT737	9.23(6.81-11.59)	9.20(6.77-11.94)	0.69
**ABL signaling**			
Nilotinib	36.16(27.32-49.48)	35.91(24.81-49.29)	0.65

Abbreviation: IC50, half maximal inhibitory concentration.
